# TD3-DLSAs: A Diffusion-Guided Low-Rank Transfer Reinforcement Learning Framework for 3D UAV Path Planning in Unknown Environments

**DOI:** 10.3390/s26217068

**Published:** 2026-11-09

**Authors:** Mingpu Huang, Hongxin Zhang, Tiezhu Zhang, Lan Bo, Haoyu Li, Mengjie Sang

**Affiliations:** 1 College of Mechanical and Electrical Engineering, Qingdao University, Qingdao 266071, China; huangmingpu@qdu.edu.cn (M.H.); bolan@qdu.edu.cn (L.B.); lihaoyu@qdu.edu.cn (H.L.); sangmengjie@qdu.edu.cn (M.S.); 2 School of Intelligent Automobile and Low-Altitude Emergency, Qingdao Heng Xing Institute of Science and Technology, Qingdao 266100, China; zhangtz@sdut.edu.cn

**Keywords:** transfer learning, diffusion model, reinforcement learning, unknown environment, path planning

## Abstract

**Highlights:**

**What are the main findings?**

- TD3-DLSAs, integrating a conditional diffusion model with LoRA-based transfer RL, achieve up to a 93% success rate in 3D mountain unknown environments and 84% in the hardest, outperforming TD3 by 24% and TD3-LSAs by 11%, while updating less than 3% of parameters for rapid adaptation.
- The diffusion-generated local subgoals reduce exploration burden, yielding higher success rates and shorter trajectories in complex terrains with low computational overhead and stable performance across large domain shifts.

**What are the implications of the main findings?**

- The synergistic combination of diffusion-based local path guidance and LoRA-enabled transfer learning provides a robust and generalizable solution for autonomous UAV navigation in completely unknown 3D environments without relying on prior map information.
- It is hoped that the TD3-DLSAs framework and the associated benchmark can offer some insights for future research on reinforcement learning-based UAV path planning, and may be extended to scenarios involving dynamic obstacles, multi-UAV coordination, sim-to-real transfer, and real-time onboard deployment.

**Abstract:**

Unmanned aerial vehicle (UAV) path planning in unknown 3D environments remains challenging due to missing prior maps, irregular terrain, and real-time obstacle avoidance requirements. This paper proposes TD3-DLSAs, a novel framework that integrates a conditional diffusion model with low-rank adaptation (LoRA) for transfer reinforcement learning. The diffusion model generates multiple feasible local subgoals based on the current UAV state, target, and local elevation map, thereby reducing the exploration burden on the reinforcement learning agent. LoRA enables rapid adaptation to new terrains by fine-tuning only a small set of low-rank matrices and scaling parameters. Extensive experiments on three increasingly difficult 3D mountain scenarios demonstrate that TD3-DLSAs achieve a success rate of 93% in the easiest and 84% in the hardest test set, outperforming standard TD3 by up to 24% and the state-of-the-art TD3-LSAs by 11%. Compared with standard RL baselines, TD3-DLSAs also tend to produce shorter and smoother trajectories in complex terrains, while maintaining a lower computational cost. These results confirm that the synergistic combination of diffusion guidance and low-rank transfer learning provides a robust and generalizable solution for autonomous UAV navigation in completely unknown environments.

## 1. Introduction

Unmanned Aerial Vehicles (UAVs) are increasingly deployed in civilian and military applications requiring autonomous navigation through complex, unstructured terrains [1,2]. Beyond navigation, UAV-based perception tasks such as small-object detection in aerial imagery [TAF-YOLO] further demand reliable autonomous flight through cluttered environments [3]. Effective path planning is critical for operational safety, energy efficiency, and mission reach [4]. Traditional planners fall into two categories: global methods such as heuristic search or stochastic sampling, which require prior environmental maps; and local reactive methods such as the dynamic window approach or artificial potential fields, which perform well in known spaces but lack long-horizon optimization [5,6,7,8]. Many real-world missions, including post-disaster search-and-rescue and surveillance in contested zones, involve environments where no prior map or obstacle database is available [9,10]. Conventional planners are inapplicable in such settings, creating a need for autonomous, map-free navigation strategies.

In this work, an unknown environment is defined as a static, unmapped operational space with a fixed goal location. The UAV has no prior terrain knowledge but carries onboard sensors, such as LiDAR or stereo vision, that provide real-time local elevation maps. The core challenge is therefore learning to navigate safely through unseen static terrain using only online sensory feedback, rather than coping with moving obstacles. Dynamic obstacles and time-varying conditions are not within the scope of this study.

Path planning for unexplored terrain must balance multiple conflicting objectives, including path length, smoothness, flight altitude, and collision safety. Multi-objective methods often require complete environmental knowledge [11,12]. Therefore, this study focuses on single-objective path planning. In this completely mapless setting, the goal is to generate a feasible and efficient route from the known starting point to the predetermined destination.

Reinforcement learning (RL) enables agents to learn reactive policies directly from high-dimensional sensory inputs. Deep RL methods, particularly off-policy algorithms such as Twin Delayed Deep Deterministic Policy Gradient (TD3), have shown success in continuous control tasks [13]. Nevertheless, applying RL to 3D UAV path planning in unknown environments faces three fundamental hurdles [14]. First, the exploration burden: the agent must learn obstacle avoidance through millions of trial-and-error interactions in a vast continuous state-action space. Second, poor generalization: a policy trained on one terrain often fails when deployed on topologically different terrains. Third, sample inefficiency: retraining from scratch for each new environment is computationally prohibitive for onboard systems. These issues severely limit the practical deployment of RL-based navigation systems [15].

Recent advances in generative modeling and parameter-efficient transfer learning offer solutions to these limitations. Diffusion models learn complex trajectory distributions through iterative denoising, outperforming variational autoencoders, which produce over-smoothed averages, and generative adversarial networks, which suffer from training instability, in path generation tasks [16,17]. Conditional diffusion models have also been applied to risk-aware decision-making and wireless trajectory optimization [18], demonstrating their versatility in sequential decision problems [19,20]. Meanwhile, Low-Rank Adaptation (LoRA) enables parameter-efficient fine-tuning by injecting trainable low-rank matrices into frozen pre-trained networks [21], reducing trainable parameters by over 95% while retaining transfer performance. While these techniques have been explored separately in autonomous driving and natural language processing, their synergistic application to UAV path planning in entirely unmapped 3D terrains remains largely unaddressed.

This paper proposes TD3-DLSAs, a framework integrating conditional diffusion guidance and a LoRA-based transfer RL. The core novelty is a closed-loop plan-act-adapt paradigm with three components. First, the diffusion model acts as an online local planner, generating multiple feasible subgoals conditioned on the current state and local elevation map, reducing the RL agent’s exploration space. Second, the TD3 policy acts as a reactive executor, tracking these subgoals while refining actions via its learned critic. Third, the LoRA module enables efficient adaptation to new terrains by updating less than 3% of network parameters, avoiding catastrophic forgetting. This integration differs from prior work that either uses diffusion for offline trajectory optimization [22], applies RL without generative guidance [23], or employs full fine-tuning, which is impractical for onboard deployment [21].

This framework is designed to reduce the actual deployment difficulty of UAVs’ autonomous navigation in unknown environments. Diffusion-generated candidate paths are filtered through a collision-checking module before subgoal selection. The LoRA-enhanced Actor-Critic architecture supports continuous 5-DOF UAV control, with a tunable scaling factor that balances frozen pre-trained knowledge and task-specific adaptation.

This work is positioned within the broader context of intelligent autonomous systems. Recent studies have advanced robust autonomous navigation under uncertainty [24] and communication-aware UAV operations, while intelligent optimization strategies have been explored for control system design [25]. Representation learning and feature-guided deep architectures have also shown promise in pattern recognition and adaptive decision-making [26,27]. However, these approaches typically assume partial environmental knowledge or rely on communication infrastructure. In contrast, our work addresses the more challenging regime of complete map-free navigation, where the agent must rely solely on onboard sensing and generative planning priors.

The main contributions of this work are threefold:

- We develop a realistic 3D simulation benchmark based on refined mountain terrain models with progressively increasing complexity, providing a challenging testbed for evaluating map-free UAV navigation.
- We propose the TD3-DLSAs framework, which for the first time integrates a conditional diffusion model as an online subgoal generator with a LoRA-enhanced TD3 agent, enabling synergistic decision-making through the “plan–act–adapt” paradigm.
- We introduce a dynamic scaling mechanism to enhance generalization ability and adaptation efficiency. Extensive experiments show that in increasingly unfamiliar 3D terrains, TD3-DLSAs achieves superior performance in terms of success rate, path smoothness, and computational efficiency.

## 2. Related Work

### 2.1. Improvements on Reinforcement Learning

Recent path planning studies increasingly combine transfer learning with deep reinforcement learning (RL) to overcome sample inefficiency and poor generalization [14,28]. Fontanesi et al. [29] applied cross-domain policy transfer, reusing navigation strategies from known terrains for map-free flight under intermittent communication. Hou et al. [30] employed curriculum-driven training, hierarchically organizing UAV control tasks from foundational maneuvers to integrated mission execution. Zu et al. [31] integrated self-imitation with transfer learning, allowing agents to refine policies using their own high-performing trajectories.

Despite these advances, existing transfer RL approaches for UAV navigation share three limitations. First, most rely on full network fine-tuning, which is computationally expensive for onboard deployment. Second, they lack explicit modeling of target-domain terrain characteristics, limiting generalization under topological shifts. Third, they do not address the exploration inefficiency in continuous 3D spaces through generative planning priors. These shortcomings motivate our integration of parameter-efficient LoRA with diffusion-based guidance.

### 2.2. Diffusion Models for Trajectory Generation

Diffusion models have emerged as powerful tools for trajectory generation. The Diffuser framework [16] integrates trajectory optimization into diffusion modeling, mitigating error accumulation in long-term predictions by learning trajectory distributions directly. Diffusion Policy [32] applies diffusion models to visuomotor control, achieving state-of-the-art performance in robotic manipulation. In the UAV domain, Drone Diffusion [18] learns quadrotor dynamics as a sequence generation task; Impedance Diffusion [33] employs image-conditioned diffusion for global UAV swarm path planning without explicit maps; and DISCO [34] applies diffusion to multi-UAV cooperative trajectory planning.

However, existing diffusion-based planners are predominantly offline or loosely coupled with RL policies—they generate complete trajectories for lower-level execution, lacking closed-loop interaction and online adaptation to environmental feedback. Their cross-domain transfer performance remains unassessed. Our work addresses this by designing the diffusion model as an online subgoal generator, tightly integrated with a LoRA-enhanced TD3 agent in a closed-loop “plan–act–adapt” paradigm.

### 2.3. Positioning and Summary

LoRA [21], originally proposed for parameter-efficient fine-tuning of large language models, has recently been explored in UAV RL. CALoRA [35] introduces an attention-enhanced LoRA variant that constructs input-dependent nonlinear adaptation manifolds, reducing initial reward variance by over 70% in AirSim. Other studies apply LoRA to federated fine-tuning [36] or decision transformer adaptation [37] for UAV control. Nevertheless, LoRA has not been systematically validated for online deep RL transfer, nor synergized with generative planning modules. This gap, along with the unresolved sample-diversity problem in target-domain replay buffers, is addressed by our framework. Table 1 positions TD3-DLSAs against representative methods.

In summary, while intelligent optimization [12], robust autonomous navigation [25], and communication-aware UAV systems [24] have advanced related domains, they typically assume partial environmental knowledge or communication infrastructure. Our work distinguishes itself by targeting complete map-free navigation through a closed-loop integration of diffusion-based planning and LoRA-based adaptation.

## 3. Materials and Methods

### 3.1. Scenario Generation

Mountainous regions represent a critical operational environment for UAVs, where the irregular terrain and altitude variations pose significant challenges in terrain comprehension. Ensuring UAV safety during flight in such uncharted settings facilitates holistic environmental perception and spatial awareness—capabilities of considerable strategic value.

**Figure 1:**
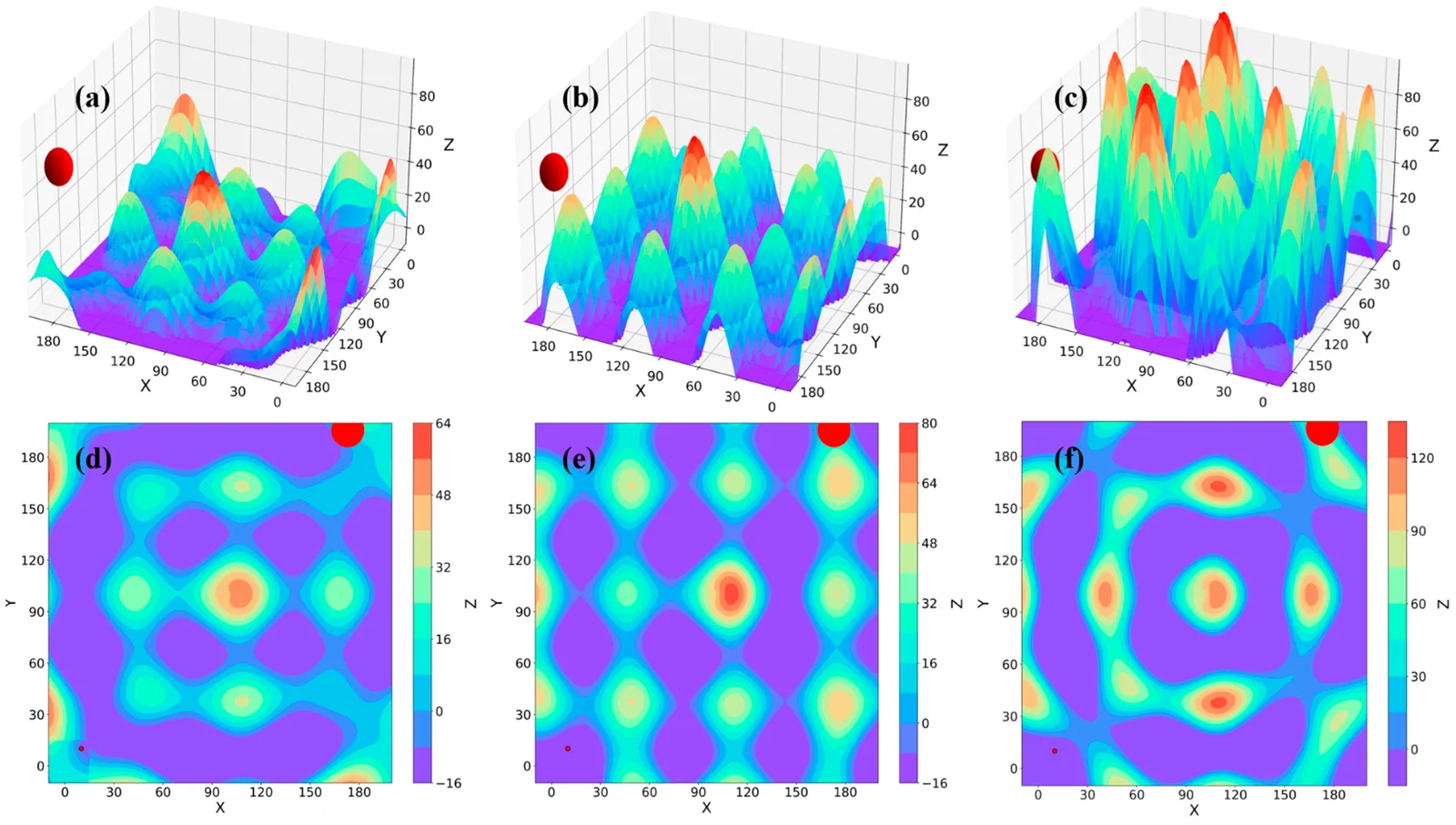
Virtual mountain environments for UAV flight experiments with increasing complexity. (**a**–**c**) 3D visualization of the terrain environments from simple to complex; (**d**–**f**) corresponding 2D contour maps of the three environments, respectively. The small red dot denotes the UAV’s take-off position, and the large red dot represents the target position. The color bar indicates the elevation value in meters.

Figure 1 presents the simulation environment adopted in this work, featuring a 3D operational space measuring 200 m × 200 m × 100 m (length × width × height). To realistically emulate complex aerial dynamics, we adopt the terrain-aware flight model proposed by Nikolos and Yu, whose formulation—given in Equation (1)—provides a physically grounded basis for evaluating navigation performance under realistic atmospheric and topographic constraints [38].

(1) $$z=\sin(y+a_{1})+a_{2}\mathrm{sinx}+a_{3}\mathrm{cosy}+a_{4}\mathrm{cosy}+a_{5}\cos\left(a_{6}\sqrt{y^{2}+x^{2}}\right)+a_{7}\sin\left(a_{7}\sqrt{y^{2}+x^{2}}\right)$$

Drawing on prior research, Equation (2) refines the initial mathematical formulation through an advanced theoretical structure and empirically calibrated parameter values. With a_1_ = 0.2 and a_5_ = 20 fixed, and a_2_, a_3_, a_4_, a_6_, a_7_, a_8_ are randomly selected once each period within the specified range. This formulation consolidates the original equations by removing redundant terms, while preserving the terrain complexity required for evaluating UAV navigation performance.

(2) $$z=\sin(y+a_{1})+a_{2}\mathrm{sinx}+a_{3}\mathrm{cosx}+a_{4}\mathrm{cosy}+a_{5}\cos\left(a_{6}\sqrt{y^{2}+x^{2}}\right)+a_{7}\sin\left(a_{8}\sqrt{y^{2}+x^{2}}\right)$$

### 3.2. Reinforcement Learning Algorithms

The Markov Decision Process (MDP) provides a rigorous mathematical framework for modeling decision-making in terrains characterized by uncertainty. The core objective of an MDP is to maximize the long-term expected return, which is exactly the same as the goal of reinforcement learning. This property simplifies state representation, while the associated policy optimization theory supplies a foundational support structure for RL algorithms. Consequently, MDPs are extensively employed as the theoretical bedrock of reinforcement learning.

An MDP is defined by the tuple {S,A,R,P,γ}, where S is the state space, A is the action space, R(s,a) is the reward function, P(s′|s,a) is the state transition probability, and γ in [0,1] is the discount factor. The goal is to find a policy π(a|s) that maximizes the expected cumulative discounted reward:

(3) $$V_{M}(s)=\sum_{a}\pi (a|s)\left[R(s,a)+\gamma \sum_{s^{\prime }}P(s^{\prime }|s,a)V_{M}(s^{\prime })\right]$$

The policy π(a|s) represents the probability of choosing action a in state s, defined as

(4) π(a|s) = P(A_t_ = a|S_t_ = s)

At each time step t, the agent in state S_t_ selects an action A_t_ according to π, transitions to S_t+1_ with probability P, and receives reward R. The overall return is the discounted sum of rewards over time.

(5) $$R_{n}=\sum_{i=n}^{N}\gamma^{i-n}\cdot r(s_{i},a_{i})$$

Here, r(s_i_,a_i_) represents the immediate reward obtained by the agent when it performs action a_i_ in state s_i_ at time step i.

Reinforcement learning necessitates the storage of extensive reward values and exhibits limited generalization capabilities. As a result, the Deep Deterministic Policy Gradient (DDPG) algorithm adopts an Actor-Critic framework wherein neural networks parameterize both the policy and value functions. Specifically, the critic network Q(s,a|θ^Q^) approximates the state–action value function, whereas the actor network μ(s|θ^μ^) directly maps states to deterministic control actions. Policy improvement is achieved by backpropagating the gradient of the expected return through the actor, following the deterministic policy gradient theorem. Equation (6) represents the gradient update formula for the actor network, while Equation (7) is utilized to update the parameters of the actor network.

(6) $$\nabla_{\theta^{\mu }}=E_{\mu }\left[\nabla_{a}Q(s,a|\theta^{Q}){|}_{s=s_{t},a=\mu (s_{t})}\nabla_{\theta^{\mu }}\mu (s|\theta^{\mu }){|}_{s=s_{t}}\right]$$

(7) $$\theta_{k+1}^{\mu }=\theta_{k}^{\mu }+\alpha E_{\mu^{\prime }}\left[\nabla_{a}Q\left(s,a|\theta_{k}^{Q}\right){|}_{a=\mu (s|\theta_{k}^{\mu })}\nabla_{\theta^{\mu }}\mu \left(s|\theta_{k}^{\mu }\right)\right]$$

The critic is updated by minimizing the temporal difference error using target networks for stability:

(8) $$y_{i}=r_{i}+\gamma \cdot Q^{\prime }\left(s_{i+1},\mu^{\prime }\left(s_{i+1}|\theta^{\mu^{\prime }}\right)|\theta^{Q^{\prime }}\right)$$

where Q′ and μ denote the target critic and actor networks, respectively. While DDPG demonstrated effectiveness in continuous control, it suffers from overestimation bias in Q-values, which can lead to suboptimal policies and training instability.

TD3 algorithm [13] addresses these limitations through three key innovations. First, TD3 utilizes two separate critic networks ($Q_{\theta_{1}}$ & $Q_{\theta_{2}}$) to produce independent Q-value estimates; the smaller of the two is then adopted when computing the target value.

(9) $$y_{i}=r_{i}+\gamma \cdot \min_{n=1,2}Q_{n^{\prime }}\left(s_{i+1},\mu^{\prime }\left(s_{i+1}|\theta^{\mu^{\prime }}\right)|\theta^{Q^{\prime }}\right)$$

This conservative update strategy mitigates Q-value overestimation. Second, TD3 introduces delayed policy updates, where the actor network is updated less frequently than the critic networks. This reduces the variance of policy updates and improves training stability. Third, TD3 adds noise *ε* to target actions during critic training:

(10) $$a^{\prime }\leftarrow \pi_{\mu^{\prime }}(s^{\prime })+\varepsilon ,\;\varepsilon \sim \mathrm{clip}\left(N(0,\sigma ),-c,c\right)$$

where a′ represents the action of the target actor network output, s′ is the next state, and *ε* denotes the added noise preventing the policy from exploiting sharp peaks in the Q-function. The target networks are updated via soft updates:

(11) $$\theta_{1^{\prime }}\leftarrow \tau \theta_{1}+(1-\tau )\theta_{1^{\prime }}$$

(12) $$\theta_{1^{\prime }}\leftarrow \tau \theta_{1}+(1-\tau )\theta_{1^{\prime }}$$

where θ_1′_, θ_2′_ are the parameter values of two target critic networks, and τ is the soft update coefficient controlling the update rate. These modifications collectively enhance the stability and sample efficiency of the algorithm, making TD3 particularly suitable for complex continuous control tasks such as UAV path planning in unknown terrains.

### 3.3. Conditional Diffusion Model (CDM)

In path planning, diffusion models are conditional probabilistic generative models that transform the trajectory generation problem into an inverse diffusion process of recovering valid trajectories from Gaussian noise [39]. We select conditional diffusion models over alternative generative approaches such as conditional variational autoencoders (CVAEs) [19] and transformer-based trajectory generators for three reasons. First, diffusion models provide stable training dynamics and avoid the posterior collapse problem that commonly affects CVAEs in high-dimensional continuous spaces. Second, the iterative denoising procedure naturally supports safety filtering at each generation step, enabling collision-free candidate paths without requiring retraining. Third, unlike autoregressive transformers that generate trajectories token-by-token, diffusion models produce full trajectories in parallel, which is critical for real-time onboard inference on resource-constrained UAV platforms [33]. During the forward diffusion phase, the model simulates trajectory degradation through environmental noise, while in the reverse phase, it learns to remove noise based on environmental constraints to generate physically feasible paths.

Assuming a UAV operates within an unknown state space ℝ^3^, let X ⊆ ℝ^3^ represent three-dimensional coordinates (x,y,z), with current position s_0_ ∈ X and target position g ∈ X. A path is represented as an ordered set P = p_0_, p_1_, …, p_L−1_ from s_0_ to g_0_, where p_i_ ∈ X.

The forward diffusion process transforms the initial path P_0_ = P into random noise P_T_ by incrementally adding Gaussian noise over T steps:

(13) $$P_{t}=\sqrt{\alpha_{t}}\cdot P_{t-1}+\sqrt{1-\alpha_{t}}\cdot \epsilon_{t}$$

where $\alpha_{t}=\sqrt{1-\beta_{t}}$, controls the noise intensity (β_t_ increases with t), and *ϵ*_t_~N(0,I_3L_) is Gaussian noise. Due to the Markov property, P_t_ can be directly expressed as

(14) $$P_{t}=\sqrt{{\bar{\alpha }}_{t}}\cdot P_{0}+\sqrt{1-{\bar{\alpha }}_{t}}\cdot \epsilon ,\;\epsilon \sim N(0,I)$$

where ${\bar{\alpha }}_{t}=\prod_{s=1}^{t}\alpha_{s}$ is the cumulative attenuation coefficient. This process simulates trajectory degradation under environmental disturbances.

The reverse diffusion process learns to recover P_t−1_ from P_t_ by modeling p(P_t−1_∣P_t_,s_0_,g,*ε*). Among them, *ε* represents the 64-dimensional elevation encoding of the local map, s_0_ represents the starting point, and g represents the destination point. Let *ϵ*_θ_(P_t_,t,s_0_,g,*ε*) denote a neural network (e.g., U-Net) that predicts noise $\hat{\epsilon }$. Then ${\hat{P}}_{t-1}$ is estimated as

(15) $${\hat{P}}_{t-1}=\frac{1}{\sqrt{\alpha_{t}}}\left(P_{t}-\frac{1-\alpha_{t}}{\sqrt{1-{\bar{\alpha }}_{t}}}\hat{\epsilon }\right)+\sigma_{t}\cdot \epsilon^{\prime }$$

where $\sigma_{t}=\sqrt{\frac{\left(1-{\bar{\alpha }}_{t-1})(1-\alpha_{t}\right)}{1-{\bar{\alpha }}_{t}}}$ and *ϵ*′~N(0,I) is a random perturbation. Starting from pure noise P_T_~N(0,I_3L_), the process iteratively generates ${\hat{P}}_{0}$ through T steps. The network *ϵ*_θ_ is trained by minimizing the noise prediction loss:

(16) $$L(\theta )=E_{t,P_{0},\epsilon_{c}}\left[{\left\|\epsilon -\epsilon_{\theta }(P_{t},t,s_{0},g,E)\right\|}_{2}^{2}\right]$$

where P_t_ is generated by Equation (14) and *ϵ*_c_ represents the actual noise. The denoiser is implemented as a U-Net with two down-sampling layers, one intermediate layer, and two up-sampling layers (channel widths 64, 128, and 256), using GroupNorm for feature channel grouping and SiLU as the activation function, consistent with the denoising network design described in Section 3.5.2. The diffusion time step t is embedded via sinusoidal positional encoding. The conditioning vector c = [E,s_0_,g] concatenates the 64-dimensional elevation encoding of the local map, the starting point, and the destination point, and is projected by a two-layer MLP to the 128-dimensional conditioning embedding before being injected into the U-Net at each resolution stage. A linear noise schedule is adopted for βt, with α_t_ = 1 − β_t_ and ${\bar{\alpha }}_{t}=\prod \left(i=1\to t\right)\alpha_{i}$, consistent with the coefficients in Equation (15), increasing from 1 × 10^−4^ to 0.02 over T = 50 training steps; at inference, the DDIM sampler accelerates the reverse process to 10 steps. The network is trained for 1500 epochs with the Adam optimizer by minimizing the noise-prediction objective defined in Equation (16). To ensure the fairness of the ablation experiments, the original training data used for TD3-DLSAs training includes all the successful paths generated by the Pre-trained model during a single training process. Each trajectory consists of 100 waypoints, which are conditionally set based on the starting position, target position, and local elevation area. These expert trajectories are collected exclusively from the source-domain pre-training process (15,000 episodes in total), in which the terrain parameters are randomly sampled within the specified ranges, thus covering a diverse set of source-domain terrain configurations. The target-domain test terrains are never involved in the diffusion-model training; consequently, no target-domain configuration is represented in the expert dataset. The complete dataset contains approximately 7000 successful trajectories in total. The loss function compels the network to learn the degradation patterns of the path under noise contamination, thereby enabling accurate noise removal during inverse diffusion and restoring the path that complies with the constraints.

During inference, we employ the denoising diffusion implicit model (DDIM) sampler to accelerate generation. Given a conditioning vector c = [x_start_,x_goal_,z_local_] containing the start position, goal position, and a local elevation patch, the model generates K = 5 candidate trajectories in parallel using 10 DDIM steps. Each candidate is then passed through a safety filtering module that checks for terrain collision at every waypoint using the local elevation map within the UAV’s onboard sensing range (R = 15 m). Among collision-free candidates, the one with the shortest path length is selected and decomposed into n(3 ≤ n ≤ 10) local subgoals uniformly spaced along the trajectory. The subgoal is updated every 10 environment steps, or immediately if the UAV deviates more than 5 m from the current subgoal. If all K candidates fail the safety check, the planner issues an unfiltered subgoal toward the goal direction as a degraded fallback; this subgoal is not collision-checked and does not guarantee safety, and obstacle avoidance in such cases relies on the reactive TD3 policy. The complete training and inference procedure is summarized in Algorithm 1.
**Algorithm 1:** The pseudo-code for the training and inference process of the Diffusion Model Path Planner**Input:** expert trajectories D, condition**Output:** local subgoals g_k_
/* Training */1: Initialize U-Net *ϵ*_θ_2: for each iteration do3:    P_0_~ D, t ~ Uniform(1…T), *ϵ*~N(0,I)4:    $P_{t}=\sqrt{{\bar{\alpha }}_{t}}\cdot P_{0}+\sqrt{1-{\bar{\alpha }}_{t}}\cdot \epsilon ,\;\epsilon \sim N(0,I)$5:    $\hat{\epsilon }$ = *ϵ*_θ_(P_t_,t,s_0_,g,*ε*)6:    $L(\theta )=E_{t,P_{0},\epsilon }\left[{\left\|\epsilon -\epsilon_{\theta }(P_{t},t,s_{0},g,E)\right\|}_{2}^{2}\right]$7: **end for**/* Inference */8: **for** i = 1 to K = 5 do9:    P_T_~N(0,I_3L_)10: **for** t = T down to 1 (DDIM, 10 steps) do11:       ${\hat{P}}_{t-1}=\frac{1}{\sqrt{\alpha_{t}}}\left(P_{t}-\frac{1-\alpha_{t}}{\sqrt{1-{\bar{\alpha }}_{t}}}\hat{\epsilon }\right)+\sigma_{t}\cdot \epsilon^{\prime }$12: **end for**13:    K candidate paths14: **end for**15: valid = collision_filter(candidate paths)16: path = select(valid)17: **return** decompose (subgoals g_K_)

### 3.4. Low-Rank Adaptation

LoRA is a parameter-efficient fine-tuning technique that injects trainable low-rank matrices into the linear layers of a pre-trained network [21]. For a pre-trained weight matrix W_0_ in ℝ^d×k^, LoRA represents the weight update as a low-rank decomposition ΔW = B × A, where A in ℝ^r×k^ and B in ℝ^d×r^ are trainable matrices with rank r < min(d,k). LoRA is applied to all linear layers of both the Actor and Critic networks. During formal training, the original parameters W_0_ are frozen, while only matrices A and B contain trainable parameters. The forward pass becomes

(17) θ_0_ + Δθ = θ_0_ + BA

During formal training, the original parameters θ_0_ ∈ ℝ^d×k^ and rank r are frozen. We can obtain the new output h:

(18) h = θ_0_x + Δθx = θ_0_x + BAx

By employing lightweight fine-tuning, this strategy effectively bridges the source and target domains, allowing for swift and precise alignment with task-specific objectives. With r = 8, the LoRA modules introduce only 2.3% trainable parameters relative to full fine-tuning, reducing GPU memory usage by approximately 40% during the adaptation phase while maintaining comparable final performance.

### 3.5. Methods

#### 3.5.1. The Restrictions on the Flight Paths of UAVs

To ensure stable and secure UAV operation, flight trajectories must adhere to kinematic constraints. Design specifications limit the maximum turning radius and climb angle:

(19) $$\theta_{\mathrm{bank}\text{-}\max}=\cos^{-1}\left(\frac{\bar{D_{i-1}D_{i}}\cdot \bar{D_{i}D_{i+1}}}{\left\|\bar{D_{i-1}D_{i}}\|\cdot \|\bar{D_{i}D_{i+1}}\right\|}\right)$$

(20) $$\psi =\arcsin\left(\frac{a_{\mathrm{vertical}}}{g}\right)$$

where D_i_ is the position vector at time i, a_Vertical_ is the vertical acceleration, g represents the gravitational acceleration of the Earth.

Equation (19) ensures the turning radius does not exceed maximum limits, while Equation (20) constrains climb angles. Additional constraints on acceleration, velocity, and step size prevent extreme conditions during training, enhancing practical flight stability.

#### 3.5.2. Model Construction and Training Parameter Configuration

In this study, a UAV is modeled as a particle operating in three-dimensional space with five degrees of freedom. This configuration enables unrestricted motion along the x, y, and z axes, as well as rotational movement in pitch and yaw. The mathematical representation is given by

(21) $$x_{t+1}=x_{t}+v_{t}\cdot \Delta T\cdot \cos(\psi_{t}+\omega_{1_{t}}\cdot \Delta T)\cdot \cos(\theta_{t}+\omega_{2_{t}}\cdot \Delta T)$$

(22) $$y_{t+1}=y_{t}+v_{t}\cdot \Delta T\cdot \sin(\psi_{t}+\omega_{1_{t}}\cdot \Delta T)\cdot \cos(\theta_{t}+\omega_{2_{t}}\cdot \Delta T)$$

(23) $$z_{t+1}=z_{t}+v_{t}\cdot \Delta T\cdot \sin(\theta_{t}+\omega_{2_{t}}\cdot \Delta T)$$

(24) $$\psi_{t+1}=\psi_{t}+\omega_{1_{t}}\cdot \Delta T,\;\theta_{t+1}=\theta_{t}+\omega_{2_{t}}\cdot \Delta T,\;v_{t+1}=v_{t}+a_{t}\cdot \Delta T$$

where (x,y,z) denote the UAV’s position in three-dimensional space; ψ and θ correspond to its yaw and pitch orientations; ω_1_ and ω_2_ are the associated angular velocities about those axes; v represents the magnitude of its translational velocity; and a signifies the linear acceleration along the direction of motion.

The observation fed to the Actor-Critic networks is an augmented state constructed by concatenating the UAV’s base state and the diffusion-generated local subgoal: $o_{t}={\left[x_{t},y_{t},z_{t},v_{t},\psi_{t},\theta_{t},d_{t},sg_{t}\right]}^{T}\in {\mathbb{R}}^{3}$, where (x_t_,y_t_,z_t_) is the current position, v_t_ is the velocity magnitude, ψ_t_ and θ_t_ are the yaw and pitch angles, D_t_ is the Euclidean distance to the target, and sg_t_ = [sg_x_, sg_y_, sg_z_] are the three-dimensional coordinates of the current diffusion-generated local subgoal in the global frame. The augmented state therefore has 10 dimensions (7 base-state + 3 subgoal), and all components are normalized to [−1, 1] before being fed to the network. The action space is a_t_ = [Δψ,Δθ,Δv] in ℝ^3^, bounded by ${\left[-a_{\max},a_{\max}\right]}^{3}$, where a_max_ = 2 rad for angular rates and 2 m/s for velocity increment. The environment time step is 0.1 s, and each episode terminates either when the UAV reaches the target (distance < 10 m), collides with terrain (z < h(x, y) + 5 m), or exceeds the maximum step count of 150. Note that the analytical height function H(x, y) is employed by the simulation environment to determine physical collision outcomes; the agent and the safety filter only have access to the locally observed elevation map within the sensing range.

The reward function employs a hybrid structure combining sparse and dense rewards [28]. Successful arrival at the target yields a terminal reward of +2000. When a collision occurs, the reward is −10 and the round ends. Additionally, a progressive navigation reward guides the agent toward the target at each step:

(25) $$R_{\mathrm{Navigation}}=\frac{D_{t-1}-D_{t}}{D_{\max}}\cdot c$$

where D_t_ and D_t−1_ are the Euclidean distances from the UAV to the goal at steps t and t − 1, respectively; D_max_ is the initial start–goal straight-line distance used to normalize the shaping term; and c = 250 is the positive reward coefficient. When the UAV approaches the goal, D_t_ < D_t−1_, so the numerator is positive and the navigation reward is positive; moving away from the goal yields a negative reward. The total reward is computed as R_t_ = R_Navigation_ + R_Arrive_ or R_Navigation_ + R_Collision_. To enhance generalization, each training episode utilizes dynamically generated map configurations with varying terrain parameters.

#### 3.5.3. Training Model

In this work, we propose a trajectory planning framework for UAVs operating in unknown environments, termed TD3-DLSAs. The framework operates in two stages: pre-training in a source domain (simplified environments with limited parameter variations) to obtain a foundational model, followed by formal training in target domains (complex unknown terrains) via transfer learning.

**Figure 2:**
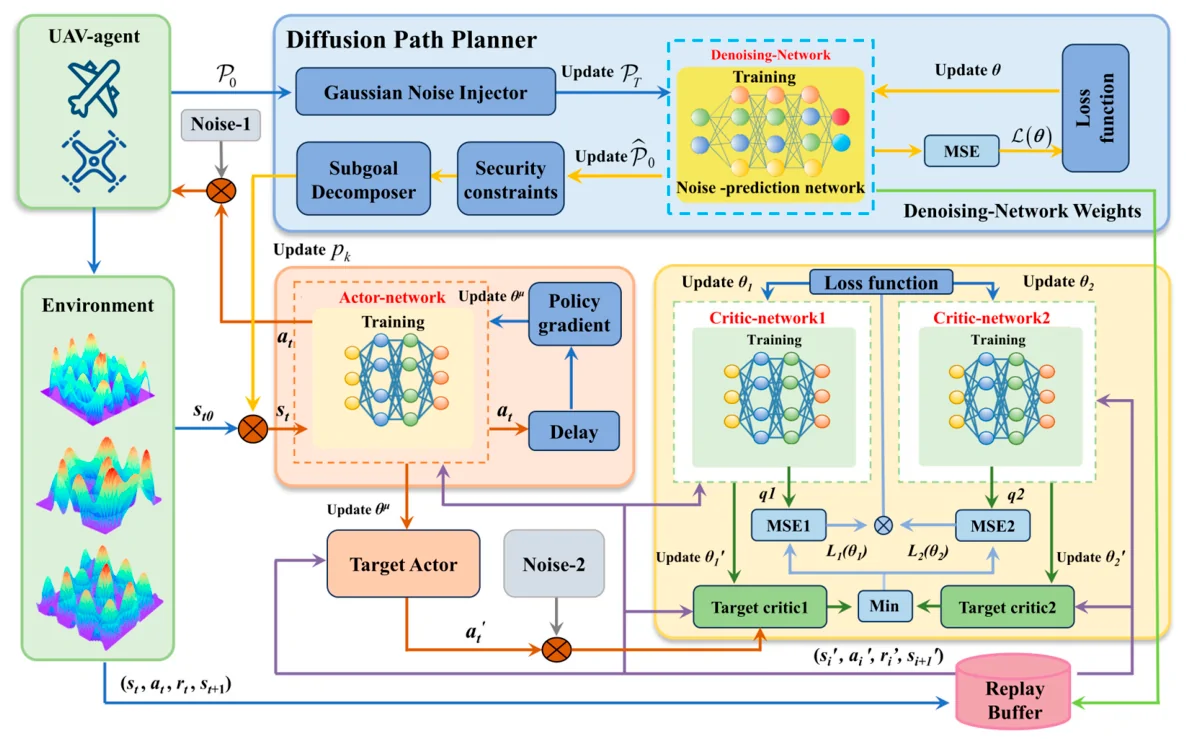
Training Framework of the TD3 Model (without the Diffusion Path Planner module) and the TD3-CDM.

**Figure 3:**
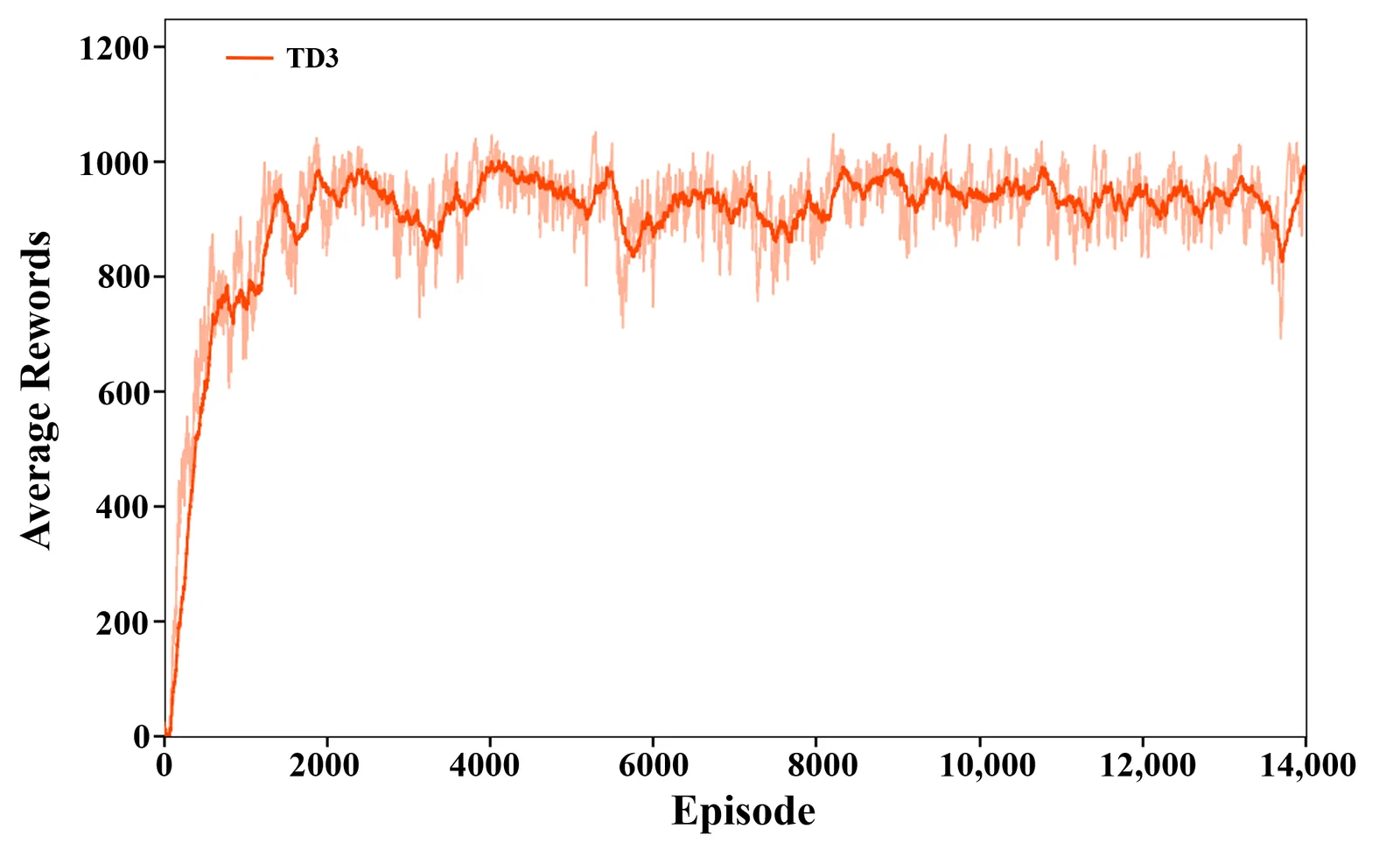
Convergence curves of the Pre-trained model (TD3).

The Pre-trained model, that is, the part in Figure 2 that does not include the “Diffusion Path Planner” module, consists of 6 components: an actor network, a target actor network, dual critic networks, and their corresponding target networks. As shown in Figure 3, the UAV acquires the environmental status. Based on the policy gradient, the action executor network will update its behavior network and the target behavior network. At the same time, the current policy is assessed by passing the selected action through the evaluation network to obtain its associated Q-value. The parameters of both the evaluation network and its target network are then updated in response to the temporal difference (TD) error.

TD3-CDM attaches a diffusion model path planner to a standard TD3 policy. The planner uses the same U-Net denoiser described in Section 3.3, with a time-embedding module, two down-sampling and two up-sampling layers, one intermediate layer, GroupNorm, and SiLU activation. Despite extensive tuning, this combination failed to achieve the desired performance in the target domain; therefore, TD3-CDM is excluded from the subsequent experiments (see Appendix A for its failure analysis, including its training curves and hyperparameters).

Pre-trained models mitigate training challenges by incrementally exposing the agent to increasingly complex unknown environments. This progressive curriculum enhances the likelihood of convergence in difficult settings and reduces overall training duration. As shown in Figure 3, the TD3 Pre-trained Model demonstrates better convergence stability. The shaded areas represent raw episode-wise reward measurements, while the smoothed curves depict the trends after applying a moving-average filter. The red curve corresponds to the convergence performance of the TD3 algorithm, which serves as the foundation for the subsequent diffusion model training.

**Figure 4:**
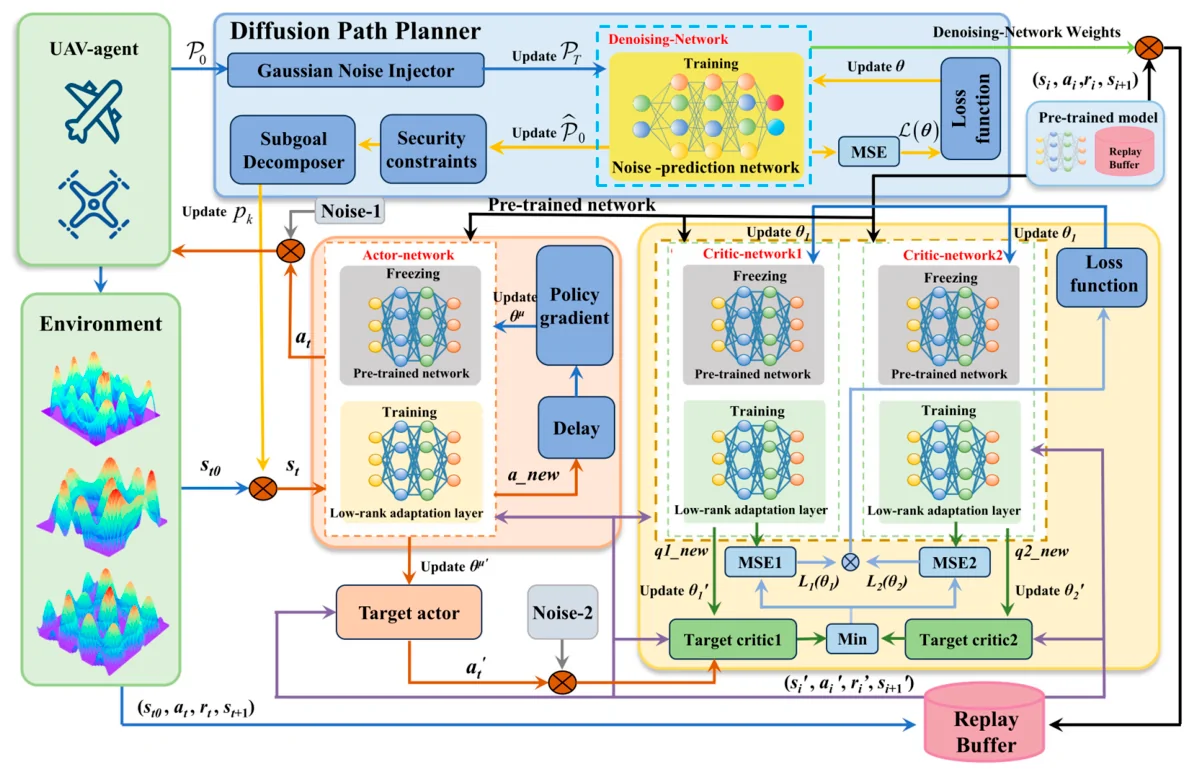
Training Framework of the formal training Model.

Through a low-rank adaptive mechanism, experiential knowledge acquired during pre-training is effectively conveyed to formal training, markedly reducing the time required for the latter. Additionally, a tunable scaling factor linked to the low-rank component is integrated into the formal training procedure, enhancing the model’s flexibility and improving the reliability of the final trained solution. The overall formal training procedure follows the pipeline depicted in Figure 4.

The formal training model adopted in this work features a seven-network architecture. This includes a denoising network, an Actor network, a target Actor network, two Critic networks, and their two target Critic networks. The denoising network adheres to the same architectural design as the TD3-CDM framework, while the remaining networks follow a unified structural configuration. Each policy network (Actor) is built upon two fundamental linear layers plus a low-rank adaptation layer, while each value network (Critic) employs two hidden linear layers followed by a scalar output layer. For the Actor, the hidden layer employs ReLU and the output layer adopts the Tanh nonlinearity to bound actions; for the Critic, all layers employ ReLU except the final linear output that produces unbounded Q-values. The low-rank adaptation module is implemented using a pair of sequential linear transformations: the first employs ReLU, and the second applies Tanh. The final output is then produced by merging the output of this adaptation layer with the pre-trained model’s output according to a specified ratio.

#### 3.5.4. Architecture Design of the Actor-Critic Networks

The Actor network consists of two fully connected layers with hidden dimension 256, each augmented with a LoRA module (Lora-a). For each linear layer with input dimension k and output dimension d, the LoRA matrices follow the standard convention A in ℝ^r×k^ and B in ℝ^d×r^; the same convention applies to every hidden layer of the Actor and Critic networks.

(26) shape(A) = (r,state_dim), shape(B) = (action_dim,r)

where state_dim = 10 is the state dimensionality, action_dim = 3 is the action dimensionality, and r = 8 is the low-rank dimension. During formal training, the pre-trained parameters remain frozen, while only the LoRA parameters A and B are updated. During the formal training process, the output of the low-rank adaptive layer after forward propagation is Δα. In one iteration of the actor network, the final output a_new_ is always described by Equation (27):

(27) a_new_ = (1 − scaling) ⋅ a + scaling⋅Δa

where scaling refers to the output ratio from the low-rank adaptation layer to the total output, which measures how much the low-rank adaptation layer influences the overall output [40]. It dynamically adjusts the actions fed into the update function, enabling precise computation of the optimization loss tailored to that particular layer [26]. The loss function for the action network is governed by Equation (28):

(28) −Q_θ_(s,a_new_)

To further refine the adaptive scaling ratio, an adaptive parameter layer is introduced into the network architecture. This layer undergoes simultaneous updates alongside matrices A and B. Through this self-tuning mechanism, the training model autonomously converges to the optimal parameter ratio, substantially boosting the effectiveness of transfer learning.

Learning of the adaptive scaling parameter. The scaling ratio s is parameterized in the log-domain, i.e., s = exp(logs), which guarantees positivity during training. It is treated as a trainable parameter and is optimized end-to-end together with the LoRA matrices A and B. For the Actor, the output of the low-rank adaptive layer is combined with the pre-trained output as a_new_ = (1 − s)a_old_ + s ⋅ a_lora_, and the scaling parameter is updated by the same actor loss defined in this section (Equation (28)) via backpropagation, with the gradient flowing through the chain:

(29) $$\frac{\partial L_{\mathrm{actor}}}{\partial \mathrm{logs}}=E\left[\frac{\partial L_{\mathrm{actor}}}{\partial a_{\mathrm{new}}}\cdot \left(a_{\mathrm{lora}}-a_{\mathrm{old}}\right)\cdot s\right]$$

For the critic, the scaling parameters of the two LoRA branches are updated through the standard TD3 critic loss function.

(30) $$L_{\mathrm{critic}}=E\left[{\left(Q-\left(r+\gamma Q^{\prime }\right)\right)}^{2}\right]$$

The learning rate of the scaling parameter is 1 × 10^−4^, smaller than the LoRA learning rate of 3 × 10^−4^, so that the ratio adapts conservatively. Through this mechanism, the model autonomously converges to the optimal balance between frozen pre-trained knowledge and task-specific adaptation.

The Critic network employs dual LoRA-enhanced branches (Lora-q) to match the TD3 twin-critic architecture. Each Critic has two hidden layers of dimension 256 and one scalar output layer. The LoRA adaptation matrices for each linear layer follow the same dimension convention as the Actor: A in ℝ^r×k^ and B in ℝ^d×r^ with r = 8.

(31) shape(A) = (state_dim+action_dim,r), shape(B) = (r,1)

During the main training phase, the pre-trained Critic network is held constant—its weights are not updated. Its input is the same Actor-generated output shared with the low-rank adaptation module. Crucially, while the Critic operates in inference-only mode, the low-rank component is fully optimized end-to-end. These two components are then merged at a predetermined ratio to produce the Lora_q output. This process yields two final Q-values, as expressed in Equation (32).

(32) q1_new_ = q1 + Δq1 ⋅ scaling_q1_, q2_new_ = q2 + Δq2 ⋅ scaling_q2_

where q1 and q2 are the frozen network’s outputs, Δq1, Δq2 come from the low-rank adaptation network. Two independent adaptation layers, whose outputs scaling_q1_ and scaling_q2_ handle parameter updates. The obtained output is fed into the loss function to calculate the target value, which is then updated via Equation (33):

(33) $$y_{i}=r_{i}+\gamma \cdot \min_{n=1,2}Q_{{\mathrm{new}}_{n}}\left(s_{i+1},\mu^{\prime }\left(s_{i+1}|\theta^{\mu^{\prime }}\right)|\theta^{Q^{\prime }}\right)$$

The overall training procedure is summarized in Algorithm 2.
**Algorithm 2:** The pseudocode of the TD3-DLSAs algorithm**Input:** start point, goal point, and sensor information**Output:** the optimal path from the start to the end1 Initialize the critic network Q_θ1_, the action network Q_θ2_, and the path generator M_Ψ_2 Initialize target networks3 Initialize experience replay buffer R4 Initialize lora matrix A, B5 Load the experience pool of the pre-trained model D6 Freeze the pretrained model7 For episode = 1 to max_episode do: a8    Initialize s9    **While** down and t < max t:10       Based on s_t0_ and D, the local path guidance p is generated through M_Ψ_        then concatenated to form s_t_.11       Generate actions a_new_ based on s_t_: a_t_ = π_ϕ_(s_t_) + Δπ_ϕ_(s_t_) + noise12       Execute action a_t_13       Observe reward rt and next state s_(t+1)0_14       Store $\left(s_{t}^{\prime },a_{t}^{\prime },r_{t}^{\prime },s_{t+1}^{\prime }\right)$ in the experience pool D’ of formal training15       s_t_ = s_t+1_16       Sample mini-batch of N transitions $\left(s_{t}^{\prime },a_{t}^{\prime },r_{t}^{\prime },s_{t+1}^{\prime }\right)$ from buffer D17       Obtain a_t+1_ = π_ϕ_(s′) + Δπ_ϕ_(s′) + noise18       Generate output of target critic network: Q_new_′ = min(q1_new_′,q2_new_′)21       Compute y_i_ by Equation (33)22       Update Lora_q networks23         **If** *t* mod *d* then:24          Update Lora_a network by the deterministic policy gradient25          Update target networks26         **End if**27    **End while**28    **End for**

## 4. Experiment and Results

### 4.1. Experimental Setup

This section delineates three experimental scenarios. The experiments conducted across these three scenarios will elucidate the correlations between algorithmic performance, environmental complexity, and source domain similarity. Specifically, formally trained models are transferred to three distinct target domains to assess the efficacy of the algorithms [31]. Notably, the similarity between these three datasets and the source domain progressively decreases, indicating a corresponding increase in scenario complexity [29]. The environmental parameters have been configured in accordance with the specifications outlined in Table 2. We compare TD3-DLSAs against several baseline and ablation algorithms (Table 3).

Hyperparameter tuning involves several critical trade-offs. First, the number of diffusion paths per iteration balances computational overhead against trajectory quality—more paths improve the chance of finding feasible routes but increase training cost. Second, path granularity (number of sampling points per path) affects both trajectory resolution and input dimensionality; finer paths provide better obstacle avoidance but increase network load. Third, the learning rate must be carefully set: too high causes instability, while too low slows convergence. Fourth, reward function design requires balancing collision penalties and altitude constraints—insufficient penalties lead to frequent collisions, while excessive ones cause overly conservative behavior. Limiting steps per episode prevents inefficient exploration while preserving adequate learning time. We provide specific hyperparameter values in Table 4 and Table 5.

To ensure reproducibility, all experiments are conducted with fixed random seeds (seed = 42 for environment initialization, seed = 123 for network parameter initialization, and seed = 456 for experience replay sampling). The hardware platform consists of an Intel Core i5-14600KF CPU and a single NVIDIA RTX 4060Ti GPU. The software environment uses Python 3.10, PyTorch 1.13.1, CUDA 11.7, and NumPy 1.23.5. All transfer-learning variants (TD3-LA, TD3-LSAs, TD3-LCs, TD3-LACs, and TD3-DLSAs) initialize from the same pre-trained TD3 backbone trained for 15,000 episodes in the source domain, ensuring a matched pretraining budget, architecture, and dataset across all transfer baselines. The standard TD3 and PPO baselines are trained from scratch in each target domain for 15,000 episodes, while all transfer variants receive an additional 15,000 episodes of target-domain adaptation. Total interaction budgets are reported explicitly to avoid misattributing pre-training cost to adaptation speed.

The three target scenarios exhibit progressively increasing domain discrepancy from the source domain. We quantify this discrepancy using the Wasserstein distance [14] between the terrain parameter distributions: Scenario 1 has a Wasserstein distance of 8.2 from the source (low discrepancy), Scenario 2 has 15.6 (medium discrepancy), and Scenario 3 has 24.3 (high discrepancy). This metric provides an explicit, quantitative measure of domain shift rather than relying solely on qualitative descriptions of terrain complexity.

Additionally, we include two further baselines that share the same pre-trained TD3 backbone and the same 15,000-episode target-domain budget as the LoRA variants. (1) TD3-FT (full fine-tuning): all network parameters of the Actor, the twin Critics, and their target networks are updated during target-domain adaptation using the same TD3 update rule and learning rate as pre-training, without LoRA or adaptive scaling; it serves as the reference for the parameter-efficiency claim, since it trains the full 5.6 M parameters instead of the 128 K updated by the LoRA variants. (2) TD3-HS (heuristic subgoal): the pre-trained TD3 policy is guided by a lightweight heuristic subgoal generator that issues a straight-line subgoal toward the goal, and switches to a tangent-direction subgoal when an obstacle is detected within the onboard sensing range (R = 15 m, forward 180° semicircle); the generated subgoal is fed to the policy in the same augmented-state form as in TD3-DLSAs, and no additional training is required. TD3-HS isolates the benefit of learning-based subgoal generation by replacing the diffusion planner with a non-learning alternative.

### 4.2. Experimental Results

For each algorithm and each scenario, a single trained policy is evaluated over 1000 test episodes under each of five evaluation seeds (10, 20, 30, 40, and 50). These evaluation seeds randomize only the unseen test terrains and the sampled start/goal pairs; they are not independent training runs, and each method–scenario policy is trained exactly once with the fixed training seeds in Section 4.1. Success rates are reported as mean with 95% confidence intervals in brackets, computed as 1.96 × SE where $\mathrm{SE}=\mathrm{std}/\sqrt{5}$ across the five seeds. Other performance metrics (smoothness, path length, and flight altitude) are reported as mean ± standard deviation. For each test episode, the terrain parameters (Equation (2)) are randomly sampled from the ranges specified in Table 2, guaranteeing that the UAV encounters unseen terrain configurations during evaluation. The following 5 metrics are recorded:

- Success rate: The percentage of test episodes in which the UAV reaches the goal region without collision. A higher success rate indicates better generalization.
- Success-Weighted Evaluation: To address the potential bias of success-only statistics, we introduce the success weighted by path length (SPL), defined as:
  (34) $$\mathrm{SPL}=\frac{1}{N}\sum_{i=1}^{N}\left[S_{i}\cdot \frac{L_{\mathrm{opt}}}{\max\left(L_{\mathrm{opt}},L_{i}\right)}\right]$$
  where S_i_ = 1 if episode i succeeds and 0 otherwise, L_opt_ = 255 m is the straight-line distance between the start and the goal, and L_i_ is the path length of episode i; failed episodes therefore contribute zero.
- Path length (m): The total distance traveled from start to goal (only successful episodes). Shorter paths indicate more efficient navigation.
- Smoothness: The average turning angle between consecutive path segments, defined as Equation (35). Lower smoothness indicates smoother trajectories.
  (35) $$\mathrm{smoothness}=\frac{\sum_{i=1}^{N-1}\cos^{-1}\left(\frac{\bar{D_{i-1}D_{i}}\cdot \bar{D_{i}D_{i+1}}}{\left\|\bar{D_{i-1}D_{i}}\|\cdot \|\bar{D_{i}D_{i+1}}\right\|}\right)}{N}$$
- Average flight altitude: The mean altitude above ground level (AGL) during successful episodes, computed as $\frac{1}{N}\sum_{i=1}^{N}(z_{i}-T(x_{i},y_{i}))$ where *N* is the number of successful episodes, *z_i_* is the UAV altitude at episode *i*, and T(*x_i_*, *y_i_*) is the terrain elevation at position (*x_i_*, *y_i_*). This metric reveals how closely the UAV follows the terrain; moderate AGL balances safety and energy.

**Figure 5:**
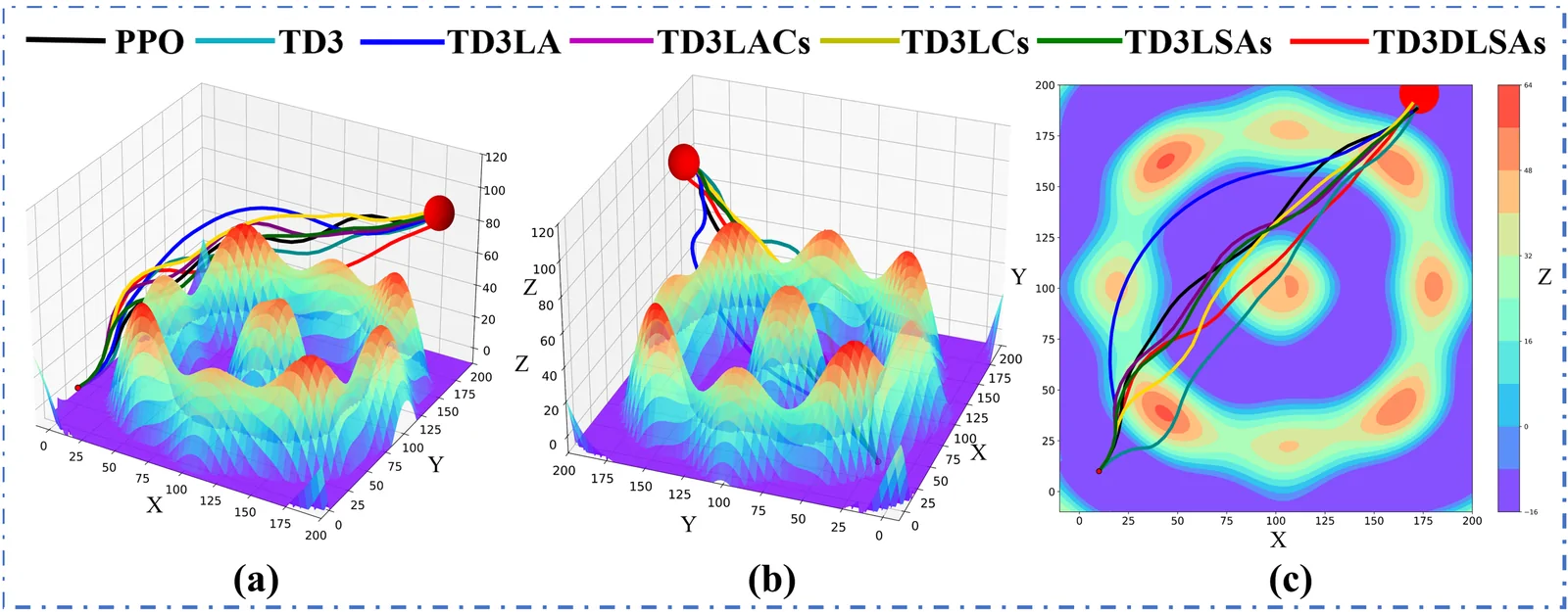
Representative trajectories generated by the seven algorithms in Scenario 1. (**a**,**b**) are three-dimensional perspective views from different angles; (**c**) is a top-down 2D view. The small red dot denotes the UAV’s take-off position, and the large red dot represents the target position.

We set the initial UAV position to (10, 10, 10) and define the target as a spherical zone centered at (175, 192, 80) with a 10-unit radius. The UAV must reach this region by following an optimal collision-free path. To compare the trajectories of the seven algorithms, Figure 5 presents results from three views: (a) and (b) are 3D views from different angles, and (c) is the top view. These visualizations highlight differences in path smoothness and obstacle avoidance.

**Figure 6:**
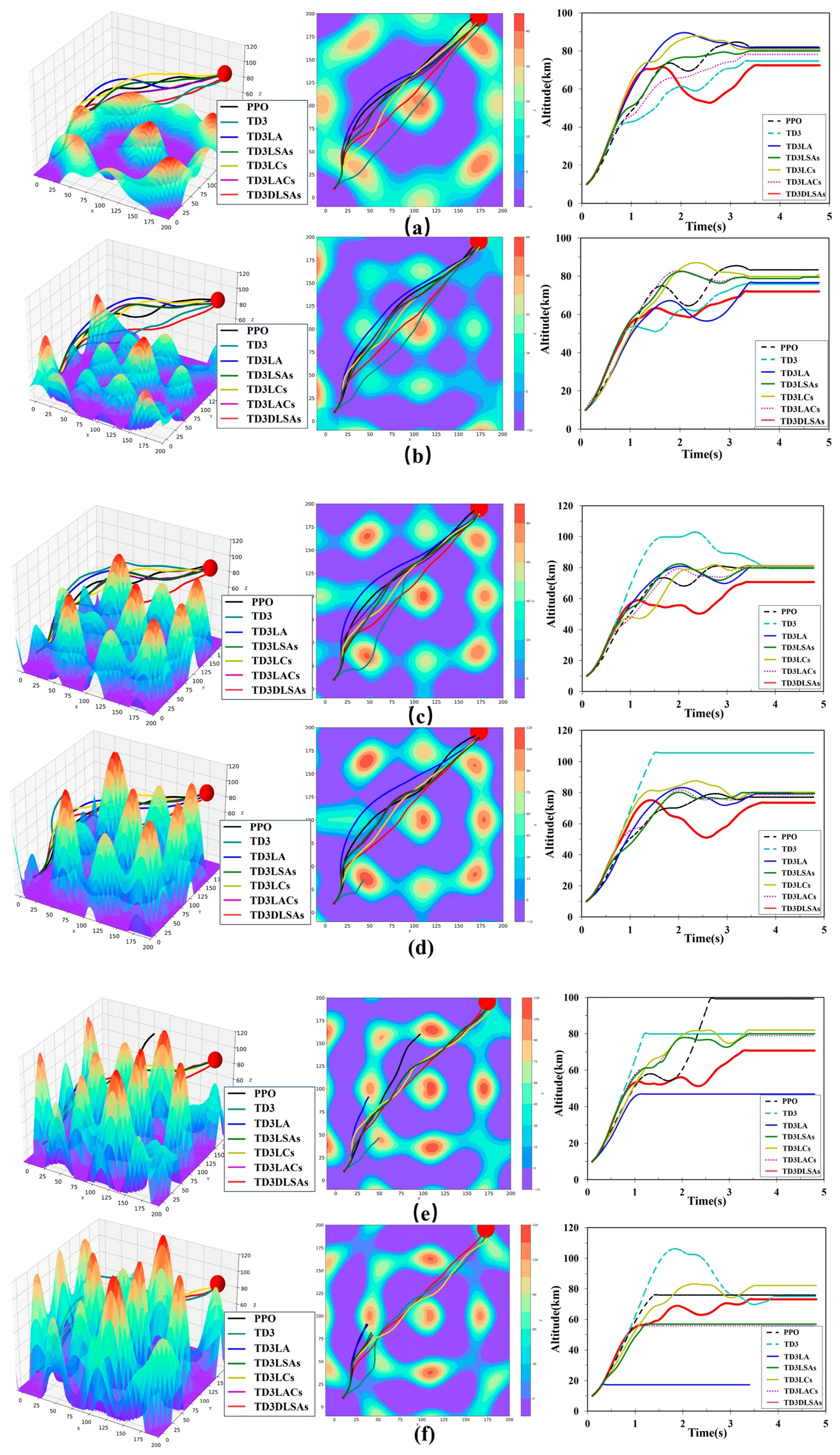
Path planning results generated by seven algorithms across three distinct scenarios. (**a**,**b**) were obtained from scenario 1. (**c**,**d**) were obtained from scenario 2. (**e**,**f**) were obtained from scenario 3.

**Figure 7:**
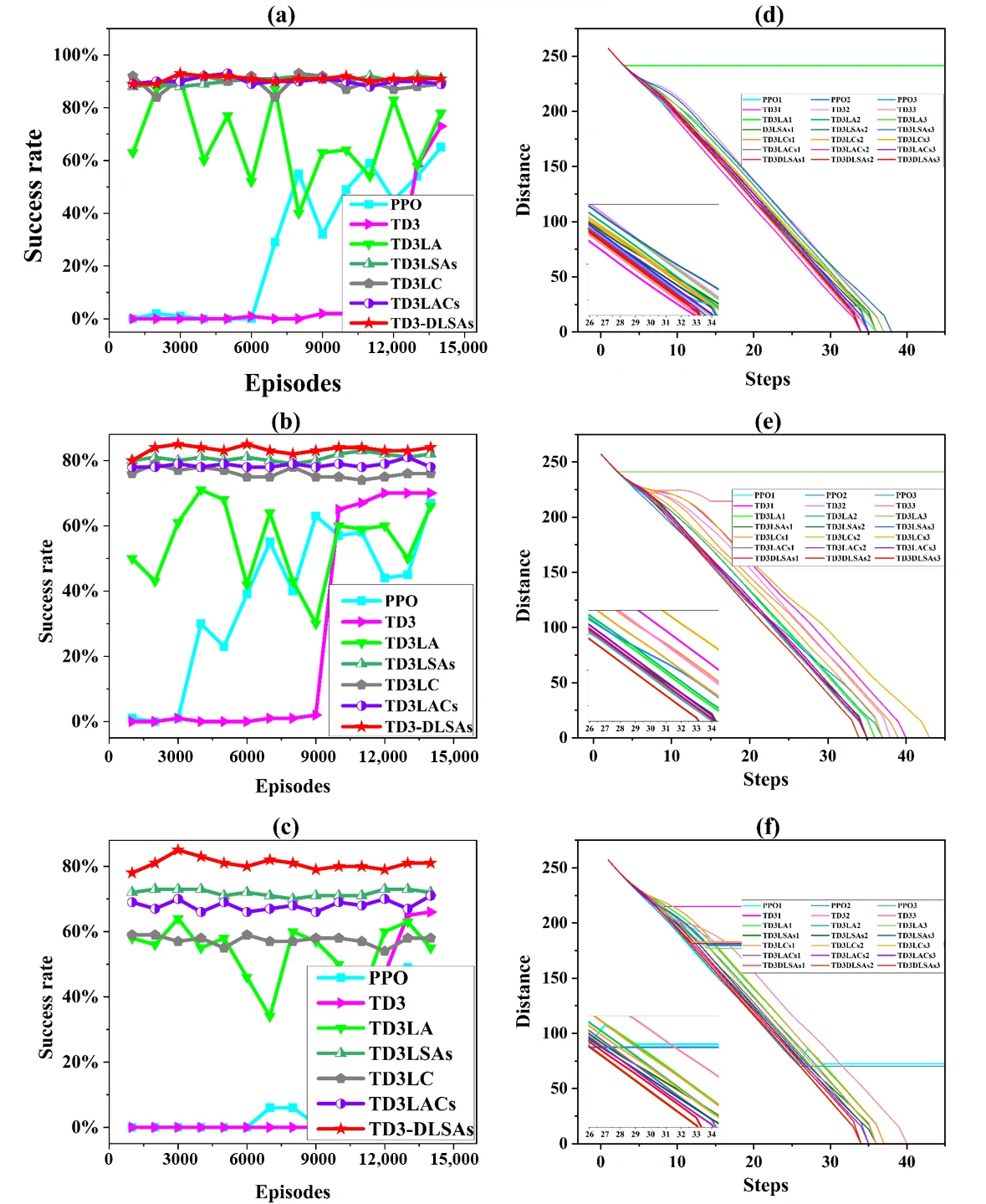
(**a**–**c**) show the success rates of the seven algorithms in three different environments; (**d**–**f**) record the distance from each step of the UAV’s flight to the goal. (The three panels correspond to Scenarios 1–3; each scenario uses the single trained policy of each method, evaluated on unseen test episodes).

Through extensive map testing, we obtained the performance data of each model and presented them in Table 6. Meanwhile, to assist readers in more intuitively observing the paths generated by each model, we generated two random visualization images and one time-domain flight altitude map for each set of environmental parameters, as shown in Figure 6. From the vertical comparison of various algorithms in Figure 7 and Table 6, we can observe that the higher the similarity between the source domain and the target domain, the higher the success rate of path planning in the test set. As the environmental complexity continuously increases, the success rates of all models will decrease accordingly, and the path length and smoothness will increase.

A comparative examination of the results in Table 6 reveals how increasing environmental complexity (from Scenario 1 to Scenario 3) affects each algorithm. As domain discrepancy grows—quantified by Wasserstein distances of 8.2, 15.6, and 24.3 from the source domain—all seven methods exhibit declining success rates. TD3-DLSAs achieve the highest success rate across all three scenarios: 93% in Scenario 1, 85% in Scenario 2, and 84% in Scenario 3. Compared with standard TD3, TD3-DLSAs improve success rates by 17, 15, and 24 percentage points in Scenarios 1, 2, and 3. Compared with TD3-LSAs, the improvement is scenario-dependent: 0% in Scenario 1 (93% vs. 93%), 3% in Scenario 2 (85% vs. 82%), and 11% in Scenario 3 (84% vs. 73%), demonstrating that diffusion guidance provides the greatest benefit in high-discrepancy environments.

PPO and TD3 represent representative on-policy and off-policy reinforcement learning baselines, respectively. As shown in Table 6, off-policy TD3 and its variants consistently outperform on-policy PPO across all three scenarios. In Scenario 3 (the hardest environment), TD3 achieves a 60% success rate compared with 40% for PPO, a 20% gap. This advantage stems from the experience replay mechanism in off-policy methods, which enables more efficient sample utilization in continuous 3D control tasks where each environment interaction is costly. Consequently, all subsequent transfer-learning variants are built upon the TD3 backbone.

To isolate the contribution of each LoRA configuration, we compare standard TD3 with four LoRA variants, as summarized in Table 6. (1) Actor-only adaptation (TD3-LA): Adding LoRA only to the actor network does not improve transfer performance; instead, success rates decrease relative to standard TD3 (74% vs. 76% in Scenario 1, 50% vs. 60% in Scenario 3), indicating that actor-only adaptation is insufficient because the critic cannot accurately evaluate actions in the new domain. (2) Critic-only adaptation (TD3-LCs): Adding LoRA only to the twin critics yields moderate improvement (90%, 78%, 69% in Scenarios 1–3), suggesting that adapting the value function is more critical than adapting the policy alone. (3) Joint adaptation with constant scaling (TD3-LACs): Applying LoRA to both actor and critics further improves performance (91%, 79%, 68%), but the constant scaling ratio limits flexibility in balancing frozen and adapted knowledge. (4) Combined with adaptive scaling joint optimization (TD3-LSAs): The introduction of a parameter adaptive scaling layer yields the best performance in the variant using only LoRA (93%, 82%, 73% respectively), and outperforms TD3-LACs by 2%, 3%, and 5% in scenarios 1, 2, and 3 respectively. This confirms that adaptive scaling contributes to effective cross-domain transfer. Notably, standard TD3 exhibits the largest performance decline from Scenario 1 to Scenario 3 (76% to 60%, a 16% drop), reflecting its sensitivity to domain shift without transfer mechanisms.

The full proposed method, TD3-DLSAs, additionally integrates a conditional diffusion model that generates local path subgoals to guide the TD3 policy. Comparing TD3-DLSAs with TD3-LSAs, which shares the same LoRA and Adaptive Scaling but lacks diffusion guidance, isolates the contribution of diffusion-generated subgoals. As shown in Table 6, the improvement is minimal in Scenario 1 (93% vs. 93%) where the terrain is simple enough that straight-line subgoals suffice, but grows to 3 percentage points in Scenario 2 (85% vs. 82%) and 11 percentage points in Scenario 3 (84% vs. 73%). This trend demonstrates that diffusion guidance provides the greatest benefit in high-discrepancy, complex terrains where naive subgoals are infeasible. Figure 7a–c presents the success-rate curves during target-domain adaptation, confirming that TD3-DLSAs consistently achieve the highest final performance across all scenarios. Figure 7d–f shows the distance-to-goal curves, confirming that TD3-DLSAs generate the most direct paths with the smallest distance oscillations, as the diffusion-generated subgoals guide the UAV toward collision-free corridors.

As shown in Table 6, the SPL ranking is fully consistent with the success-rate ranking in all three scenarios, confirming that the conclusions drawn from Table 6 are not an artifact of success-only statistics. Because failures are penalized with zero SPL, methods with lower success rates are substantially depressed: in Scenario 3, PPO and TD3-LA achieve SPL values of 0.31 and 0.44, respectively, whereas TD3-DLSAs reaches 0.80, an improvement of 0.3 over standard TD3 and 0.12 over TD3-LSAs. The only inversion occurs in Scenario 1, where TD3-LSAs (0.90) slightly outperforms TD3-DLSAs (0.88); both methods achieve the same success rate (93%), while TD3-LSAs produces slightly shorter paths (263 m vs. 269 m), which is consistent with the trade-off discussed in Section 5.2. In the harder scenarios, where navigation efficiency matters most, TD3-DLSAs attain the highest SPL in both Scenario 2 (0.81 vs. 0.78) and Scenario 3 (0.80 vs. 0.68), indicating that diffusion-guided subgoals improve not only the success rate but also the efficiency of successful navigation.

To evaluate the fallback behavior, we additionally logged fallback events during the test phase of TD3-DLSAs. The fallback is triggered only when all K = 5 diffusion-generated candidates fail the safety check, in which case a straight-line subgoal toward the goal is issued and subsequently executed by the trained TD3 policy with reactive obstacle avoidance. In Scenario 1, the fallback was triggered in approximately 3% of test episodes; the collision rate of these episodes was about 30%, and about 70% of them still reached the goal. In Scenario 2, the trigger rate was approximately 5%, with a post-fallback collision rate of about 34% and a success rate of about 66%. In the hardest scenario (Scenario 3), the trigger rate increased to approximately 7%, the post-fallback collision rate remained at about 40%, and about 60% of the episodes involving fallback were still completed successfully. The fallback is activated rarely (approximately 3–7% of test episodes) and only in the most difficult situations where no diffusion candidate passes the safety check. We emphasize that this fallback is **not a safety guarantee**: its subgoal is not collision-checked, and the 30–40% collision rate within fallback episodes shows that these episodes remain high-risk; collision avoidance in such episodes relies entirely on the reactive TD3 policy. Because fallback episodes account for only a small fraction of the test set, the fallback contributes at most approximately 3 percentage points to the overall failure rate. Its only role is to provide a degraded direction when no validated candidate exists, rather than to ensure a safe maneuver; a dedicated safety layer or emergency maneuver for such all-candidate-rejected cases remains future work.

To substantiate the efficiency claim of LoRA (Section 3.5) and the benefit of learned subgoal generation, we add two further baselines (Table 7). TD3-FT performs full fine-tuning of all network parameters under the same training budget, serving as the direct reference for the parameter-efficiency claim: it updates the full 5.6 M parameters instead of the 128 K (2.3%) updated by the LoRA variants, whereas the LoRA variants reduce adaptation-phase GPU memory by approximately 40% relative to full fine-tuning (Section 5.3, Table 8). Despite this efficiency advantage, TD3-FT achieves success rates of 93%, 83%, and 73% in Scenarios 1–3, which are comparable to those of the strongest LoRA variant without diffusion (TD3-LSAs: 93%, 82%, 73%; within 1 percentage point in Scenario 2), confirming that LoRA attains competitive performance at a fraction of the parameter budget. TD3-HS replaces the diffusion planner with a simple heuristic that issues a straight-line subgoal toward the goal, deviating to the tangent direction only when an obstacle is detected within the sensing range; its success rates of 78%, 71%, and 60% are only marginally higher than those of standard TD3 (76%, 70.1%, 60%) in Scenarios 1 and 2 and identical in Scenario 3, and are markedly lower than those of TD3-DLSAs in the harder scenarios (60% vs. 84% in Scenario 3), demonstrating that naive heuristic subgoals provide little guidance and that learned diffusion subgoals are essential in complex terrains. The third baseline, TD3-CDM (TD3 with diffusion but without LoRA), failed to achieve the expected performance in the target domains; its training curves, hyperparameters, and failure characteristics are reported in Appendix A.

**Figure 8:**
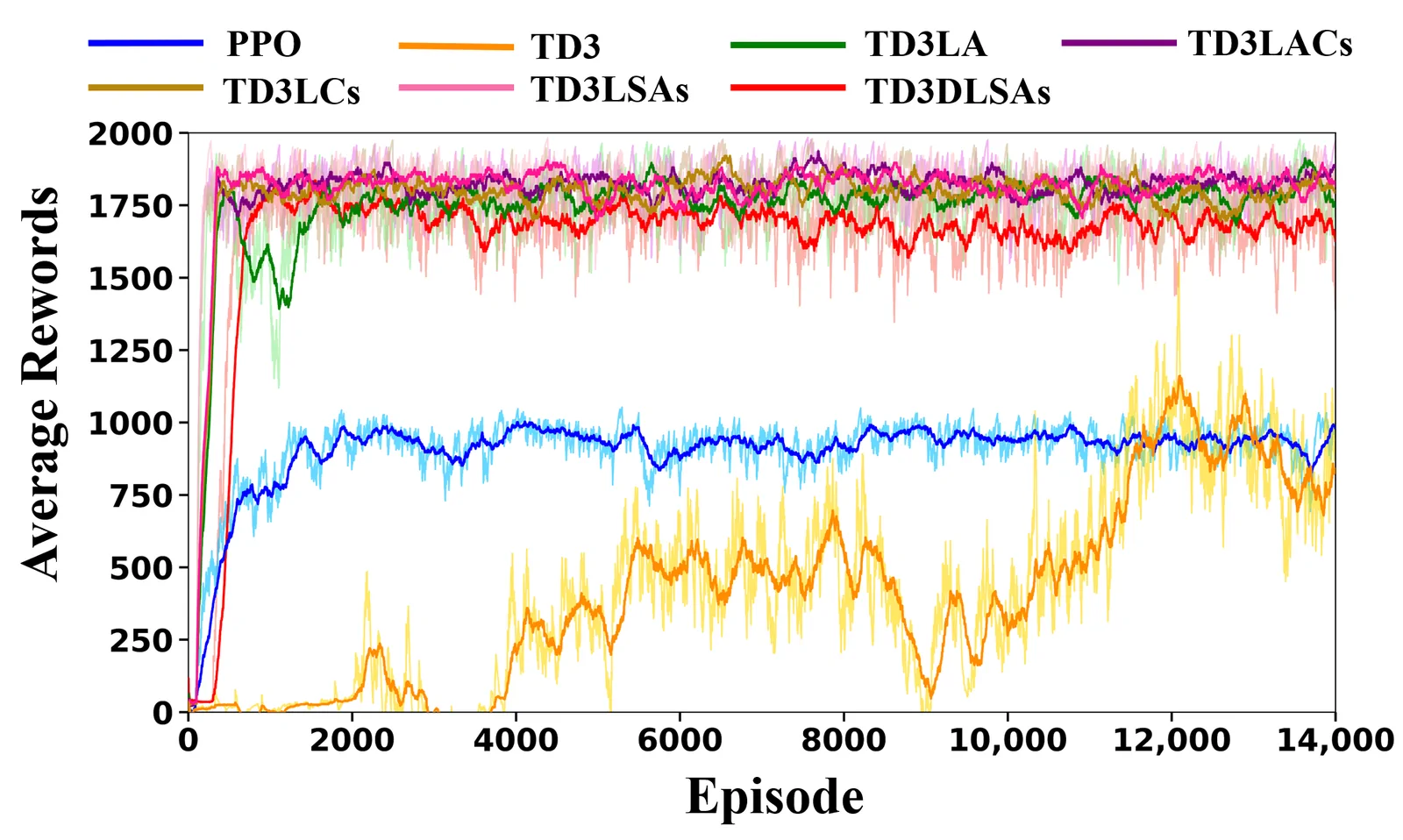
The training convergence performance of the 7 algorithms under the initial environmental parameters. The light-colored curves represent the raw training rewards without exponential smoothing, while the solid darker curves show the smoothed convergence trends.

We carefully logged the rewards throughout training to evaluate the convergence behavior of all seven algorithms (Figure 8). The transfer-learning variants benefit from pre-trained initialization and reach high reward levels rapidly, while standard TD3 and PPO require more episodes to converge from scratch. It is important to distinguish two aspects of efficiency: (1) parameter efficiency, where LoRA reduces trainable parameters to 2.3% of full fine-tuning, lowering memory usage and per-step computation; and (2) final performance, where TD3-DLSAs achieve the highest success rates across all scenarios. However, among the LoRA-based transfer variants, TD3-DLSAs do not converge significantly faster than TD3-LSAs or TD3-LACs in terms of training episodes; their primary advantage lies in higher final success rates, especially in Scenario 3, rather than faster convergence. This distinction clarifies that the computational efficiency claim refers to parameter and memory efficiency, not necessarily fewer training episodes to convergence.

Ablation study: To isolate the individual contribution of each proposed component, we compare the seven variants across the three scenarios. (1) LoRA adaptation: Comparing standard TD3 with TD3-LA (actor-only LoRA) and TD3-LCs (critic-only LoRA) reveals that critic-only adaptation is more effective than actor-only adaptation (90% vs. 74% in Scenario 1), while both underperform joint adaptation. (2) Adaptive scaling: TD3-LSAs (adaptive scaling) outperform TD3-LACs (constant scaling) by 2, 3, and 5 percentage points in Scenarios 1, 2, and 3, respectively, confirming that dynamic parameter adaptation improves transfer performance. (3) Diffusion guidance: Comparing TD3-DLSAs with TD3-LSAs isolates the effect of diffusion-generated subgoals. The improvement is minimal in Scenario 1 (0 percentage points, 93% vs. 93%) where straight-line subgoals suffice, but grows to 3 percentage points in Scenario 2 (85% vs. 82%) and 11 percentage points in Scenario 3 (84% vs. 73%), demonstrating that diffusion guidance provides the greatest benefit in high-discrepancy, complex terrains. This ablation confirms that the performance gains of TD3-DLSAs originate primarily from the synergistic combination of diffusion guidance and LoRA adaptation, with diffusion guidance being the dominant factor in the hardest scenario.

Evaluation methodology note. Path length, smoothness, and average flight altitude are computed over successful episodes only. To avoid success-only bias, we additionally report SPL (Equation (34)), which assigns zero to failed episodes. The straight-line distance from start to goal (~255 m) serves as a lower bound; the best methods achieve 263–269 m, only 3–6% above this optimum.

### 4.3. Generalization on Real Digital Elevation Model

**Figure 9:**
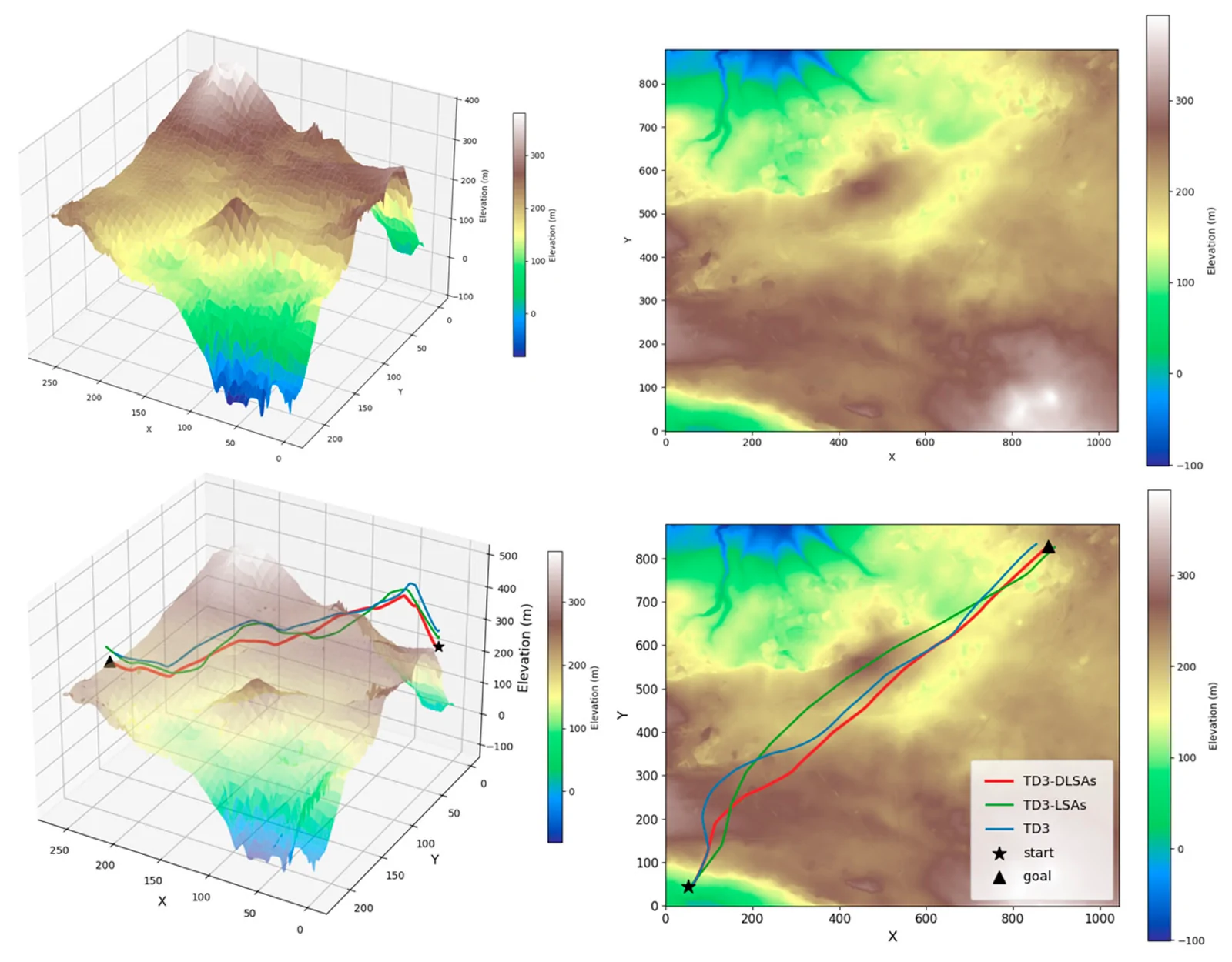
S4 real-DEM generalization scenario: (**top row**) 3D and 2D views of the ChrismasTerrain elevation; (**bottom row**) representative trajectories of TD3-DLSAs (red), TD3-LSAs (green), and TD3 (blue) from start (★) to goal (▲).

To evaluate cross-environment generalization under realistic sensing conditions, we introduce a fourth scenario (S4) using the ChrismasTerrain real LiDAR digital elevation model (5 m resolution). As shown in Figure 9, this terrain features rugged mountain ridges and deep valleys, topographically distinct from the smooth sinusoidal terrains used in training. The DEM is resized to 200 m × 200 m to match the simulation grid. In each episode, the start (★) and goal (▲) positions are randomly sampled, and the UAV is never retrained on this terrain. We run 100 independent episodes per method. 

As shown in Table 9, without noise, TD3-DLSAs achieve 90% success, outperforming TD3-LSAs (83%) and vanilla TD3 (55%). This confirms that the diffusion-generated subgoals transfer to unseen, geographically distinct terrain without additional training. The 35-percentage-point gap between TD3-DLSAs and vanilla TD3 highlights that the learned subgoal prior is the primary source of generalization.

Under sensor noise, all methods degrade, but the ranking is preserved and the gap widens. TD3-DLSAs drops by 5 percentage points (90% → 85%), TD3-LSAs by 10 points (83% → 73%), and vanilla TD3 by 13 points (55% → 42%). The diffusion planner degrades more gracefully because it conditions subgoals on a learned trajectory distribution rather than reacting directly to every noisy observation—effectively acting as a temporal smoother against sensory uncertainty. In contrast, vanilla TD3 maps raw (noisy) observations directly to low-level control commands, making it highly sensitive to per-step estimation errors.

**Figure 10:**
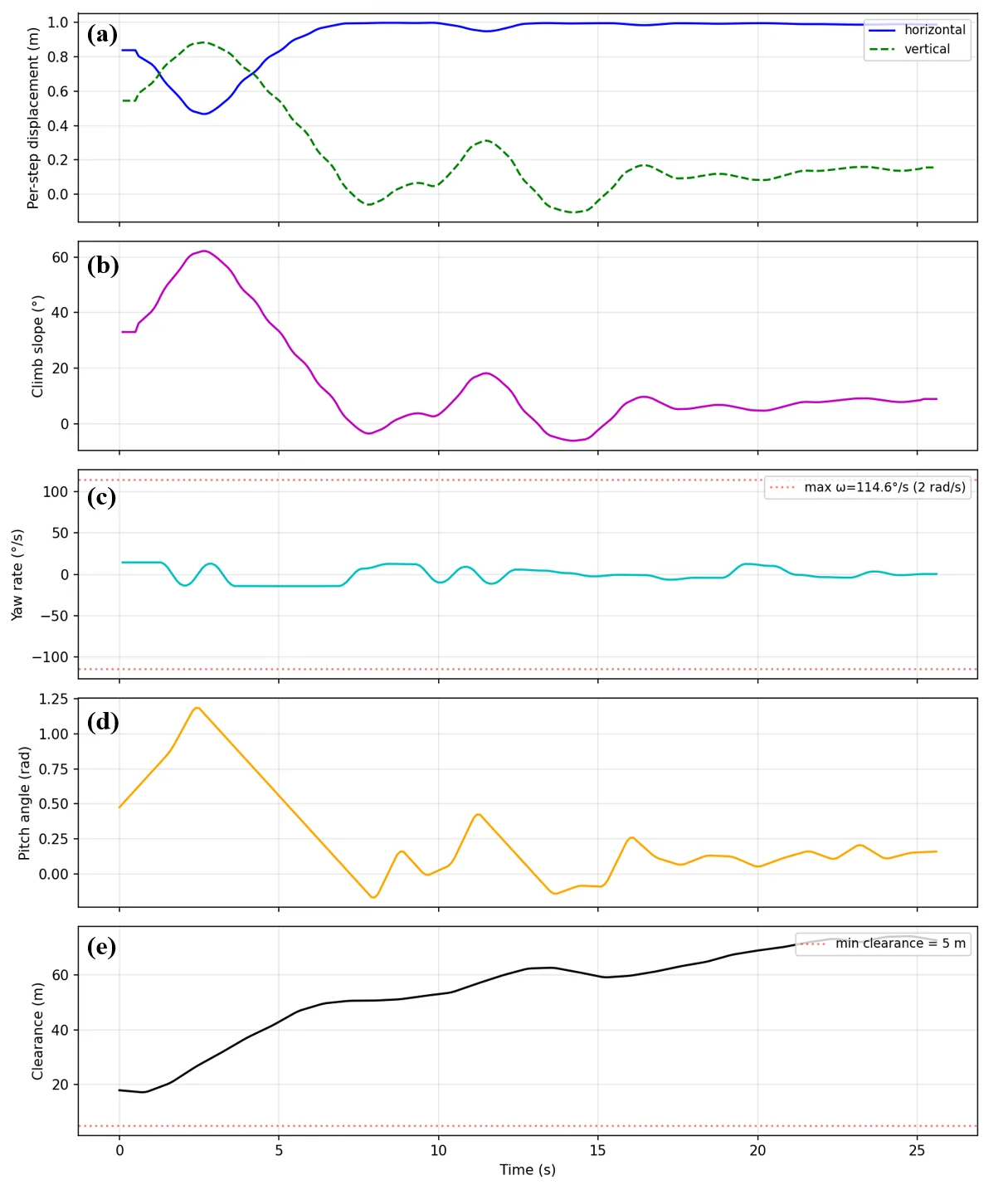
Flight feasibility time series of a representative successful TD3-DLSAs episode on the ChrismasTerrain real LiDAR DEM: (**a**) horizontal and vertical step displacement along the resampled trajectory; (**b**) trajectory climb slope arctan (Δz/Δh); (**c**) yaw rate against the ±2 rad/s (±114.6°/s) bound; (**d**) body pitch angle; (**e**) terrain clearance against the 5 m safety threshold. The time axis is re-parameterized along the recorded arc length at a representative reference cruise speed of 10 m/s; panels (**b**–**e**) verify the angular velocity and altitude restrictions that were set.

To verify that the generated trajectories respect the kinematic constraints defined in Section 3.5 (angular rates bounded by ±2 rad/s, Δt = 0.1 s, minimum terrain clearance 5 m), we record a representative successful episode and plot the per-step displacement, climb angle, yaw rate, pitch angle, and terrain clearance in Figure 10. All yaw-rate commands remain within the ±114.6°/s bound, and minimum clearance stays above 17 m throughout the flight.

## 5. Discussion

We have restructured the Section 5 into three parts. The first part explains the principles and limitations of low-rank adaptation and transfer learning. The second part details how the diffusion model improves performance, especially in hard scenarios. The third part presents limitations and future work.

### 5.1. Mechanisms and Limitations of Low-Rank Adaptation and Transfer Learning

Low-rank adaptation (LoRA) is a parameter-efficient fine-tuning technique that injects trainable low-rank matrices into the network layers. In our TD3-DLSAs framework, with rank r = 8 and scaling factor α = 16, LoRA reduces the number of trainable parameters to only 2.3% of full fine-tuning (128 K out of 5.6 M total parameters), while preserving the representational power of the original Actor and Critic networks. This parameter reduction translates to approximately 40% less GPU memory usage during the adaptation phase and lower per-step computation overhead, enabling faster adaptation to new environmental features without suffering from catastrophic forgetting. Transfer learning from a pre-trained TD3 backbone further provides a strong initialization, as the model already captures generic path-planning priors from the source domain.

However, these components have inherent shortcomings. LoRA, by constraining weight updates to a low-rank subspace, may limit the model’s capacity to learn highly complex, non-linear decision boundaries in extremely cluttered terrains [41]. Transfer learning, while beneficial, can also lead to negative transfer when the source and target environments have very different topological structures. Indeed, our results in

Table 6 show that TD3-LSAs (without diffusion) suffer a 20% drop in success rate from Scenario 1 to Scenario 3, indicating that pure low-rank or transfer approaches are insufficient for robust generalization.

### 5.2. How Diffusion Guidance Enhances Performance in Challenging Scenarios

The diffusion model in our method addresses the above limitations by providing explicit geometric guidance. Unlike standard RL policies that learn a mapping from states to actions purely through trial and error, the diffusion model is trained to generate feasible local subgoals given the current UAV state and a terrain heightmap. It does so by progressively denoising a randomly sampled Gaussian distribution into a plausible waypoint, conditioned on the environment context.

In easy terrains (Scenario 1), the straight-line subgoal is often sufficient, and the RL agent can learn obstacle avoidance without diffusion guidance. Indeed, TD3-DLSAs and TD3-LSAs achieve identical success rates in Scenario 1 (93% vs. 93%), and TD3-LSAs even produce slightly shorter paths (263 m vs. 269 m) and (0.12 vs. 0.14) in this scenario. However, when terrain complexity increases (e.g., high peaks blocking the direct path in Scenario 3), the diffusion model can propose alternative directions that circumvent large obstacles—acting as a planning heuristic. Even if the generated subgoal is not globally optimal, it provides a feasible local direction that the RL agent can refine using its learned critic. This synergy explains why TD3-DLSAs outperforms TD3-LSAs (which uses LoRA and Adaptive scaling but no diffusion) by 0, 3, and 11 percentage points in Scenarios 1, 2, and 3, respectively, and reduces path length by 10 m in the hardest scenario (268 m vs. 278 m). This improvement comes with a trade-off: TD3-LSAs achieves lower smoothness values (i.e., smoother trajectories) across all three scenarios (0.12, 0.11, 0.13 vs. 0.14, 0.14, 0.14), as the diffusion-generated subgoals occasionally introduce sharper direction changes. The ablation analysis confirms that the performance gain in hard scenarios originates primarily from diffusion guidance rather than LoRA adaptation: comparing TD3-LSAs (LoRA + Adaptive scaling, no diffusion) with TD3-DLSAs (full method) isolates the diffusion contribution to 11 percentage points in Scenario 3, while LoRA adaptation alone (TD3-LA vs. TD3) provides inconsistent benefits across scenarios. The diffusion guidance effectively compensates for the representational constraints of low-rank adaptation and mitigates negative transfer, leading to robust performance across varying domain similarities.

### 5.3. Limitations and Future Directions

Limitations:

- Computational overhead and model complexity: We report detailed computational metrics for deployment on embedded UAV hardware. The diffusion model adds approximately 15 ms per subgoal regeneration on the RTX 4060 (measured as wall-clock inference time for generating 5 candidate trajectories with 10 DDIM steps), while the TD3 policy forward pass requires less than 1 ms. The peak latency of the regeneration frame is therefore approximately 16 ms (a worst-case upper bound of ~63 Hz); since the subgoal is reused for the following 10 environment steps (Section 3.3), the amortized average latency per decision step is approximately 2.5 ms (Table 8). Memory consumption: the full TD3-DLSAs model occupies 40.2 MB of disk space and 1.1 GB of GPU VRAM during inference, compared with 12.6 MB and 0.4 GB for standard TD3. The LoRA modules account for only 2.3% of trainable parameters, reducing adaptation-phase GPU memory by approximately 40% relative to full fine-tuning. This overhead is suitable for surveillance and search-and-rescue UAVs but may be excessive for racing drones.
- Benchmark limitations: While Scenarios 1–3 use smooth synthetic sinusoidal terrains, we additionally evaluate on the ChrismasTerrain real LiDAR DEM (5 m resolution) in Section 4.3, where TD3-DLSAs achieves 90% success without retraining. However, both the synthetic and real DEM terrains are represented as single-valued elevation grids (no overhangs, bridges, or vertical cliffs); future work should include multi-valued environments with vegetation and urban structures. Future benchmarks should include multi-valued terrain, vegetation, and urban structures.
- Static environment assumption: Our method assumes the terrain is static. Dynamic obstacles (e.g., moving UAVs) are not considered. Extending to dynamic environments would require the diffusion model to condition on predicted obstacle trajectories.
- Simulation-to-reality gap: The UAV is modeled as a 5-DOF kinematic point mass rather than a full dynamical model with mass, thrust, drag, and actuator constraints. Although Section 4.3 verifies trajectory feasibility under the prescribed ±2 rad/s angular-rate and 5 m clearance constraints on real elevation data, hardware-in-the-loop or real-flight validation with sensor noise and actuator dynamics remains future work. Domain randomization during training could further close this gap.

Future work:

- Adaptive diffusion update frequency: Dynamically extend the subgoal reuse interval beyond the fixed L = 10 steps when the UAV remains close to the current subgoal, reducing computational load [16].
- Latent diffusion models: Compress path representations into a latent space to accelerate inference.
- Real-world experiments: We acknowledge that this study is limited to simulation-based validation, as our current laboratory conditions do not permit real-world UAV experiments under extreme terrains. Future work will focus on sim-to-real transfer by deploying the TD3-DLSAs framework on embedded platforms (e.g., NVIDIA Jetson, PX4-based UAVs) and addressing the domain gap through domain randomization and hardware-in-the-loop testing.
- Multi-UAV coordination: Extend the framework to cooperative path planning for multiple UAVs, where the diffusion model generates collision-free subgoals for each agent [42].

## 6. Conclusions

This paper presented TD3-DLSAs, a diffusion-guided low-rank transfer reinforcement learning framework for 3D UAV path planning in unknown environments. The key idea is to use a conditional diffusion model to generate local subgoals online, which are then fed as augmented state inputs to a LoRA-enhanced TD3 agent. The agent is pre-trained in a source domain and then efficiently fine-tuned in the target domain by updating only low-rank adaptation parameters.

Extensive experiments on three challenging 3D terrain scenarios demonstrated that:

- TD3-DLSAs achieves a success rate of 93% in easy scenarios and 84% in the hardest scenario, outperforming standard TD3 by 17, 15, and 24 percentage points in Scenarios 1, 2, and 3, respectively (up to 24% in the hardest scenario). Compared with TD3-LSAs (the strongest baseline without diffusion guidance), the improvement is scenario-dependent: 0 percentage points in Scenario 1, 3 percentage points in Scenario 2, and 11 percentage points in Scenario 3, demonstrating that diffusion guidance provides the greatest benefit in high-discrepancy, complex terrains.
- The diffusion guidance significantly improves success rate in complex terrains (up to 11 percentage points in Scenario 3) and reduces path length by 10 m in the hardest scenario, albeit with a slight smoothness trade-off compared with TD3-LSAs. LoRA enables parameter-efficient adaptation with only 2.3% trainable parameters and approximately 40% less GPU memory relative to full fine-tuning.
- Ablation studies confirm that (1) joint actor-critic LoRA with adaptive scaling (TD3-LSAs) outperforms actor-only, critic-only, and constant-scaling variants; (2) diffusion guidance (TD3-DLSAs vs. TD3-LSAs) contributes 0, 3, and 11 percentage points in Scenarios 1, 2, and 3, respectively, with the largest contribution in the hardest scenario; and (3) the combination of diffusion guidance and LoRA adaptation yields synergistic performance gains that neither component achieves alone.

Our work offers a practical solution for autonomous UAV navigation in unknown static 3D environments. We acknowledge that the current evaluation is limited to synthetic single-valued terrain with simplified UAV kinematics and no dynamic obstacles, and that real-world deployment will require validation on physical platforms with more realistic dynamics and sensor noise. Future work will extend the framework to dynamic environments, multi-UAV coordination, and sim-to-real transfer on embedded UAV hardware.

## Figures and Tables

**Table 1:** Comparison of TD3-DLSAs with representative UAV navigation methods.

Method	Environment	Planning Strategy	Transfer	Param. Efficient	Closed-Loop
Standard TD3	3D static	RL only	No	—	—
Diffuser	2D/3D	Offline diffusion	No	—	No
Diffusion Policy	2D/3D	Offline diffusion + RL	No	—	No
Drone Diffusion	3D	Diffusion + controller	No	—	Partial
CALoRA	3D	RL only	LoRA	Yes	—
TD3-DLSAs (Ours)	3D	Diffusion + RL	LoRA	Yes (<3%)	Yes

**Table 2:** Terrain parameter ranges for the three test scenarios.

Parameter	Scenario 1 (Easy)	Scenario 2 (Medium)	Scenario 3 (Hard)
a_2_	[2,25]	[2,30]	[2,35]
a_3_	[2,30]	[2,40]	[2,40]
a_4_	[2,20]	[2,20]	[2,25]
a_6_	[0.6,1.0]	[0.6,1.1]	[0.6,1.1]
a_7_	[15,30]	[12,35]	[2,40]
a_8_	[0.6,1.2]	[0.6,1.2]	[0.6,1.2]

**Table 3:** Algorithm acronyms and their corresponding meanings.

Algorithm	Description
PPO	On-Policy RL algorithm with clipped surrogate objective.
TD3	Standard Off-Policy algorithm without transfer learning.
TD3-LA	TD3 with LoRA only on the actor network.
TD3-LCs	TD3 with LoRA only on the critic networks (two critics).
TD3-LACs	TD3 with LoRA on both actor and critics, but without adaptive scaling.
TD3-LSAs	TD3 with LoRA on both actor and critic, plus adaptive scaling, no diffusion model.
TD3-DLSAs	Full proposed method.

**Table 4:** Hyperparameters for reinforcement learning.

Parameter	Value
Discount factor	0.99
Target network update rate	0.005
Policy noise	0.1
Noise clip	0.1
Policy update delay	2
Actor learning rate	3 × 10^−4^
Critic learning rate	3 × 10^−4^
LoRA learning rate	3 × 10^−4^
Scaling parameter learning rate	1 × 10^−4^
Batch size N	256
Replay buffer size	1 × 10^6^

**Table 5:** Hyperparameters for Diffusion Model Path Planner.

Parameter	Value
Number of diffusion steps T (training)	50
Number of inference steps (DDIM)	10
Noise schedule	Linear from 10^−4^ to 0.02
Number of candidate paths K	5
Output path length L (waypoints)	10
U-Net channels	64,128,256
Conditioning embedding dimension	128
Training epochs	1500

**Table 6:** Experimental Results of Seven Algorithms. Success rates are reported as mean ± 95% confidence interval across the five evaluation seeds and 1000 test episodes per condition; the seeds randomize only the test terrains and start/goal pairs and do not correspond to independent training runs. Other metrics are reported as mean ± standard deviation. SPL is defined in Section 4.2 (Equation (34)); failed episodes contribute zero to SPL.

Scenario	Algorithm	Smoothness (−)	Length (m)	Success Rate (95% CI)	Success-Weighted Evaluation	Avg. Flight Alt. (m)
	PPO	0.14 ± 0.03	303 ± 12.6	71 ± 1.0%	0.6	59 ± 8.2
	TD3	0.14 ± 0.02	275 ± 10.2	76 ± 0.3%	0.7	66 ± 8.1
	TD3-LA	0.14 ± 0.02	273 ± 10.3	74 ± 0.2%	0.69	55 ± 7.0
1	TD3-LCs	0.15 ± 0.02	280 ± 10.1	90 ± 0.3%	0.82	65 ± 8.3
	TD3-LACs	0.13 ± 0.02	272 ± 9.3	91 ± 0.2%	0.83	62 ± 7.6
	TD3-LSAs	0.12 ± 0.02	263 ± 8.5	93 ± 0.2%	0.9	62 ± 7.4
	TD3-DLSAs	0.14 ± 0.03	269 ± 10.1	93 ± 1.0%	0.88	52 ± 8.1
	PPO	0.15 ± 0.03	305 ± 14.2	56 ± 1.2%	0.47	59 ± 8.8
	TD3	0.15 ± 0.03	272 ± 11.8	70.1 ± 0.5%	0.65	66 ± 8.5
	TD3-LA	0.11 ± 0.02	287 ± 11.1	69 ± 0.4%	0.61	55 ± 7.2
2	TD3-LCs	0.16 ± 0.02	304 ± 11	78 ± 0.4%	0.65	65 ± 8.0
	TD3-LACs	0.15 ± 0.02	280 ± 10.8	79 ± 0.3%	0.72	62 ± 7.9
	TD3-LSAs	0.11 ± 0.02	271 ± 9.9	82 ± 0.3%	0.78	61 ± 7.5
	TD3-DLSAs	0.14 ± 0.02	269 ± 10.5	85 ± 1.1%	0.81	51 ± 8.2
	PPO	0.12 ± 0.03	336 ± 18.1	40 ± 1.6%	0.31	60 ± 8.7
	TD3	0.17 ± 0.04	304 ± 16.2	60 ± 1.6%	0.5	68 ± 8.4
	TD3-LA	0.11 ± 0.03	288 ± 13.6	50 ± 1.3%	0.44	55 ± 7.3
3	TD3-LCs	0.16 ± 0.03	304 ± 13.3	69 ± 1.2%	0.58	65 ± 7.5
	TD3-LACs	0.15 ± 0.03	280 ± 13.1	68 ± 0.9%	0.62	62 ± 8
	TD3-LSAs	0.13 ± 0.03	278 ± 13.1	73 ± 0.9%	0.68	62 ± 7.6
	TD3-DLSAs	0.14 ± 0.03	268 ± 14.6	84 ± 1.1%	0.8	53 ± 8.2

**Table 7:** Additional transfer-learning baselines. TD3-FT fine-tunes all network parameters during target-domain adaptation; TD3-HS guides the pre-trained TD3 policy with a heuristic subgoal generator. Both share the pre-trained backbone and target-domain budget of the LoRA variants.

Algorithm	S1 Succ. (95% CI)	S1 SPL	S2 Succ. (95% CI)	S2 SPL	S3 Succ. (95% CI)	S3 SPL
TD3-FT	93 ± 0.2%	0.92	83 ± 0.3%	0.8	73 ± 0.8%	0.69
TD3-HS	78 ± 0.3%	0.77	71 ± 0.5%	0.66	60 ± 1.2%	0.52

**Table 8:** Computational cost comparison. Inference latency is measured on a single RTX 4060; the average is amortized over the subgoal update interval L = 10 (Section 3.3), computed with the policy forward pass at its 1 ms upper bound. All diffusion-free algorithms share a peak latency of <1 ms, a model size of 12.6 MB, and 0.4 GB inference VRAM, with the heuristic rules of TD3-HS adding negligible computation; TD3-DLSAs differs only in its diffusion planner: 40.2 MB, 1.1 GB VRAM, a peak of ~16 ms in the regeneration frame (5 candidates × 10 DDIM steps, ~15 ms, plus <1 ms policy), and ~2.5 ms average.

Algorithm	Trainable Params	Peak (ms/Frame)	Avg. (ms/Step)	Model Size (MB)	VRAM (GB)
PPO, TD3	5.6 M (100%)	<1	<1	12.6	0.4
TD3-FT	5.6 M (100%)	<1	<1	12.6	0.4
TD3-HS	0 (no training)	<1	<1	12.6	0.4
TD3-LA, LCs, LACs, LSAs	128 K (2.3%)	<1	<1	≈12.6	0.4
TD3-DLSAs	128 K (2.3%)	~16	~2.5	40.2	1.1

**Table 9:** Real-DEM generalization results (100 episodes per method, random start/goal pairs).

Algorithm	Clean Success	+Sensor Noise	Drop
TD3	55%	42%	13%
TD3-LSAs	83%	73%	10%
TD3-DLSAs	90%	85%	5%

Sensor noise model: The same Gaussian noise is injected at every control step for all three methods to ensure a fair comparison: position estimate noise σ_p_ = 0.5 m (consistent with GPS/RTK-grade IMU drift for low-altitude flight) and local elevation-map noise σ_h_ = 0.2 m (consistent with short-range LiDAR point-cloud dispersion). These magnitudes follow the measurement-noise specifications reported in prior UAV terrain-mapping work.

**Figure A1:**
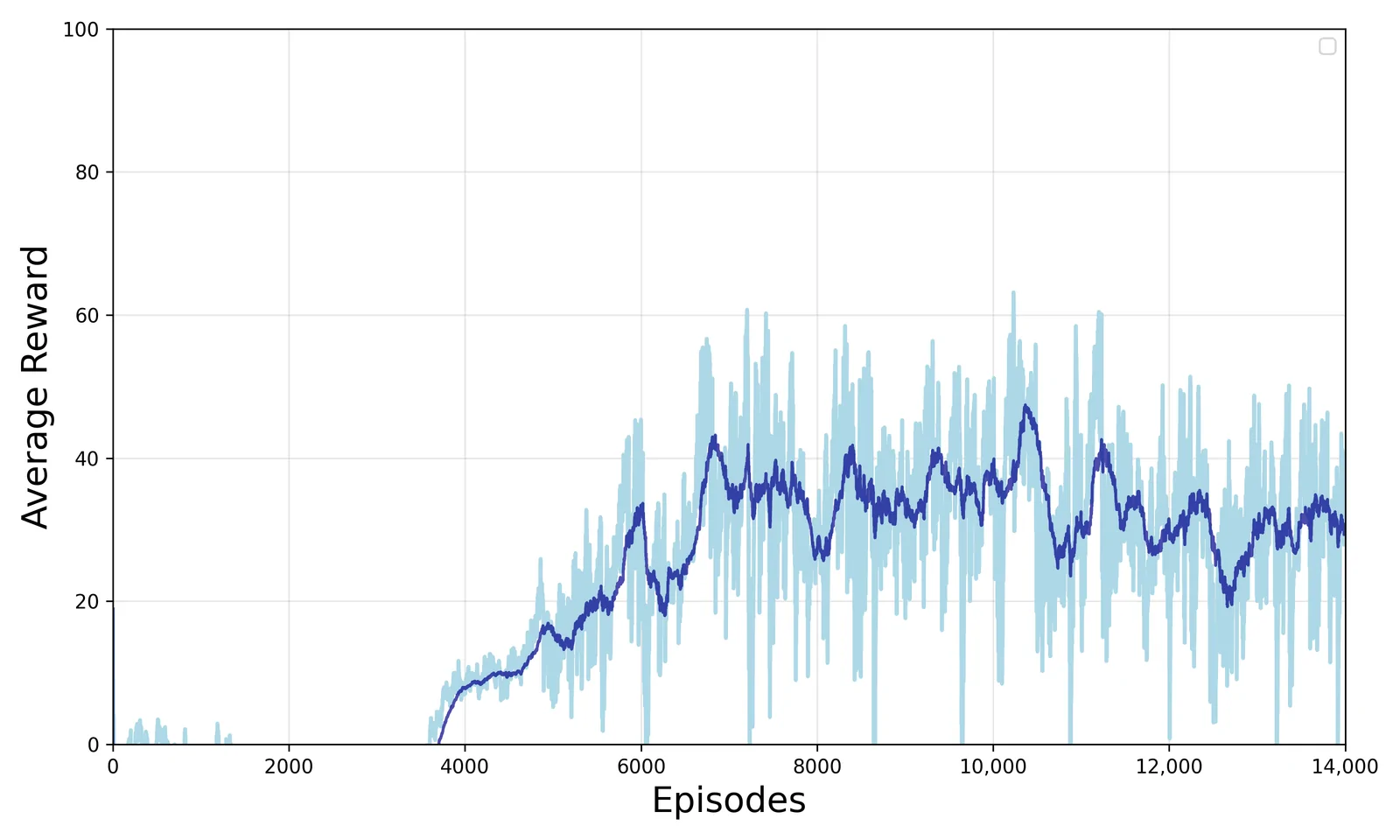
Average episode reward of TD3-CDM during target-domain training. The reward stays near zero for the first 3000 episodes, rises to approximately 42 around episodes 6000–7000, and then plateaus at 30–35 with high variance without further improvement.

## Data Availability

The original contributions presented in this study are included in the article. Further inquiries can be directed to the corresponding author.

## Abbreviations

The following abbreviations are used in this manuscript:

UAV	Unmanned Aerial Vehicle
RL	Reinforcement Learning
DRL	Deep Reinforcement Learning
TD3	Twin Delayed Deep Deterministic Policy Gradient
DDPG	Deep Deterministic Policy Gradient
PPO	Proximal Policy Optimization
SAC	Soft Actor-Critic
LoRA	Low-Rank Adaptation
CDM	Conditional Diffusion Model
DDPM	Denoising Diffusion Probabilistic Models
VAE	Variational Autoencoder
CVAE	Conditional Variational Autoencoder
GAN	Generative Adversarial Network
MDP	Markov Decision Process
TD	Temporal Difference
CNN	Convolutional Neural Network
MLP	Multi-Layer Perceptron
ReLU	Rectified Linear Unit
Adam	Adaptive Moment Estimation
GPU	Graphics Processing Unit
CPU	Central Processing Unit
ISAC	Integrated Sensing and Communication
APF	Artificial Potential Field
SPL	Success weighted by Path Length
AGL	Above Ground Level
CI	Confidence Interval
SE	Standard Error
std	Standard Deviation
ICML	International Conference on Machine Learning
IROS	Intelligent Robots and Systems
NeurIPS	Neural Information Processing Systems

## Appendix A

TD3-CDM is constructed by attaching the conditional diffusion planner of Section 3.3 to a standard TD3 policy, without the LoRA-based transfer mechanism of Section 3.5. Its input formulation is identical to that of TD3-DLSAs: the diffusion planner is pre-trained in the source domain and frozen during target-domain adaptation, and every L = 10 environment steps, it regenerates K = 5 candidate subgoal paths, filters them with the same terrain collision rule (z < h(x, y) + 5 m), and feeds the first valid subgoal to the policy in the augmented-state form. The only difference from TD3-DLSAs is that the TD3 policy is trained from scratch in each target domain: it neither loads the pre-trained backbone nor updates any LoRA modules. Apart from this missing pre-trained backbone and LoRA adaptation, TD3-CDM shares the same TD3 configuration and the same 15,000-episode target-domain budget as the from-scratch TD3 baseline, so that its performance difference can be attributed to the transfer mechanism rather than to any training-setting mismatch.

The TD3 hyperparameters follow Table 5 (γ = 0.99, τ = 0.005, policy noise 0.1, noise clip 0.1, policy update delay 2, learning rate 3 × 10^−4^, batch size 256, replay buffer 10^6^), and the diffusion settings are the same as in Section 3.3 (T = 50, DDIM 10 steps, K = 5, L = 10); the exploration noise starts at 0.15 and decays exponentially with a factor of 0.995 per 100 episodes down to a floor of 0.14. Apart from the absence of the pre-trained backbone and LoRA modules, TD3-CDM uses the same batch size, 15,000-episode training budget, and all remaining TD3 hyperparameters as the from-scratch TD3 baseline in Section 4.1, so that the two differ only in whether source-domain transfer is provided. Despite extensive tuning, TD3-CDM did not reach competitive performance. As shown in Figure A1, the average episode reward stays near zero for the first 3000 episodes, rises to approximately 42 around episodes 6000–7000, and then plateaus at 30–35 with high variance, without further improvement over the full 15,000-episode budget. That is, the agent learns only a primitive obstacle-avoidance behavior early in training and then fails to close the remaining gap toward reliable goal reaching.

We attribute this failure to the absence of source-domain knowledge transfer rather than to the diffusion planner itself. First, the diffusion-generated subgoals are conditioned on source-domain trajectory statistics, whereas a randomly initialized policy has no prior on the control and environment dynamics of the target domain; the two action distributions are initially inconsistent, so the subgoal guidance cannot be converted into consistent action corrections. Second, the critics provide unreliable value estimates on the augmented state space during early training, which introduces high noise into the target values and destabilizes the policy gradient. Third, the exploration noise decays before the policy has learned to follow the subgoals, trapping the agent in a locally suboptimal policy. These factors jointly prevent TD3-CDM from exploiting the diffusion guidance, confirming that the pre-trained backbone and the LoRA adaptation are prerequisites for diffusion-guided transfer in this framework, and justifying its exclusion from the main comparisons in Section 3.5.2.
